# Asymmetric regulation of quorum-sensing receptors drives autoinducer-specific gene expression programs in *Vibrio cholerae*

**DOI:** 10.1371/journal.pgen.1006826

**Published:** 2017-05-26

**Authors:** Amanda Hurley, Bonnie L. Bassler

**Affiliations:** 1 Department of Molecular Biology, Princeton University, Princeton, NJ, United States of America; 2 Howard Hughes Medical Institute, Chevy Chase, MD, United States of America; Max Planck Institute for Terrestrial Microbiology, GERMANY

## Abstract

Quorum sensing (QS) is a mechanism of chemical communication that bacteria use to monitor cell-population density and coordinate group behaviors. QS relies on the production, detection, and group-wide response to extracellular signal molecules called autoinducers. *Vibrio cholera*e employs parallel QS circuits that converge into a shared signaling pathway. At high cell density, the CqsS and LuxPQ QS receptors detect the intra-genus and inter-species autoinducers CAI-1 and AI-2, respectively, to repress virulence factor production and biofilm formation. We show that positive feedback, mediated by the QS pathway, increases CqsS but not LuxQ levels during the transition into QS-mode, which amplifies the CAI-1 input into the pathway relative to the AI-2 input. Asymmetric feedback on CqsS enables responses exclusively to the CAI-1 autoinducer. Because CqsS exhibits the dominant QS signaling role in *V*. *cholerae*, agonism of CqsS with synthetic compounds could be used to control pathogenicity and host dispersal. We identify nine compounds that share no structural similarity to CAI-1, yet potently agonize CqsS via inhibition of CqsS autokinase activity.

## Author summary

Bacteria communicate, count their numbers, and act as collectives using a process called quorum sensing. Quorum sensing relies on the production, release and group-wide detection of small molecules called autoinducers. Curiously, quorum-sensing bacteria often employ multiple autoinducers that funnel information into a common signal transduction pathway. This feature of quorum-sensing systems has made it difficult to understand if or how individual autoinducers could produce unique quorum-sensing responses making the benefit of using multiple autoinducers mysterious. The global pathogen *Vibrio cholerae* uses two, seemingly redundant, quorum-sensing autoinducers to control pathogenicity. Here, we discover a mechanism that drives an increase in production of one autoinducer receptor, but not the other. This asymmetry allows *V*. *cholerae* to disproportionately respond to one autoinducer over the other autoinducer, and in so doing, control behaviors exclusively in response to the dominant autoinducer. Our work solves the issues of whether and how *V*. *cholerae* achieves autoinducer-specific control of quorum-sensing behaviors. In this manuscript, we also identify nine synthetic compounds that potently mimic the dominant *V*. *cholerae* autoinducer. These compounds represent leads for *V*. *cholerae* prophylactics and/or therapeutics.

## Introduction

Quorum sensing (QS) is a cell-cell communication process that enables bacteria to monitor population density and control behaviors as collectives. QS relies on the production, release, accumulation, and detection of extracellular signal molecules called autoinducers. At low cell density (LCD), when autoinducer concentration is low, bacteria carry out behaviors that are successful when they act as individuals. At high cell density (HCD), in response to autoinducer accumulation and detection, bacteria launch programs of gene expression that underlie group behaviors. Often, QS systems integrate information encoded in multiple autoinducers, which presumably enables bacteria to monitor numbers of kin (intra-species QS), related family members (intra-genera QS), and non-kin (inter-species QS) in the vicinal community [1,2].

**Fig 1:**
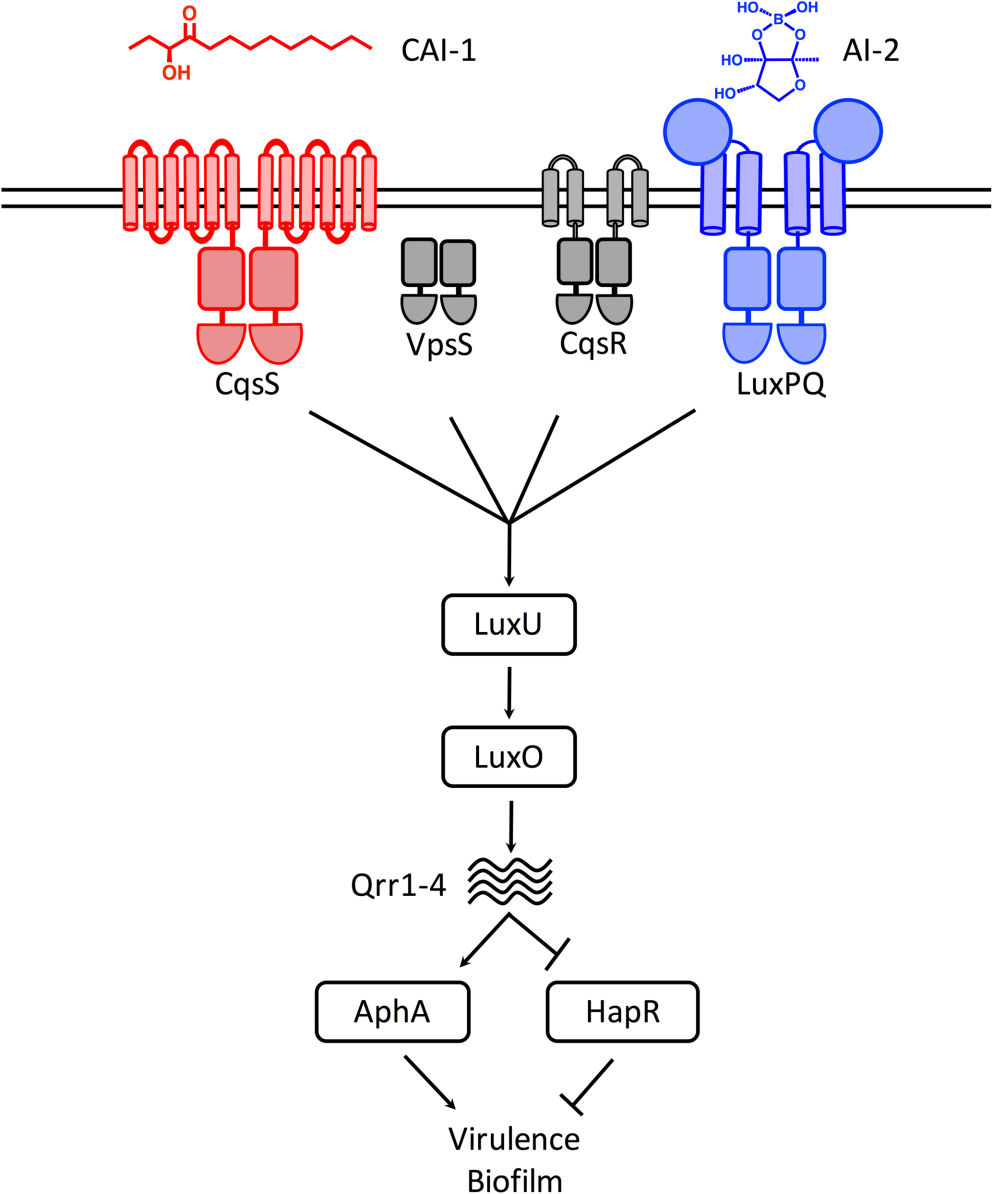
Simplified *V*. *cholerae* QS circuit. At LCD, the transmembrane receptors CqsS (red) and LuxPQ (blue) act as kinases that autophosphorylate and transfer phosphate, via LuxU, to LuxO. LuxO~P activates transcription of genes encoding the Qrr1-4 sRNAs. Qrr1-4 activate translation of AphA and repress translation of HapR, which are, respectively, the major LCD and HCD transcription factors. At HCD, in the presence of the CAI-1 and AI-2 autoinducers, CqsS and LuxPQ are inhibited for kinase activity so they act as phosphatases that strip phosphate from LuxO via LuxU. Dephosphorylated LuxO is inactive, Qrr1-4 sRNA are not transcribed, and therefore, HapR protein is produced while AphA protein is not. HapR represses virulence factor production and biofilm formation. The figure shows the direction of phosphoryl flow in the LCD state. The CAI-1 and AI-2 structures are shown. VpsS and CqsR (gray) are two newly discovered receptors that act in parallel to CqsS and LuxPQ. The VpsS and CqsR ligands are not known.

The pathogen and model QS bacterium *Vibrio cholerae* has two known QS autoinducers: cholera autoinducer-1 (CAI-1, (*S*)-3-hydroxytridecan-4-one) [3] and autoinducer-2 (AI-2, (2*S*, 4*S*)-2-methyl-2,3,3,4-tetrahydroxytetrahydrofuran borate) [4] (Fig 1). CAI-1 and AI-2 are produced by the CqsA [5] and LuxS [6,7] synthases and detected by the trans-membrane bound histidine sensor kinase receptors CqsS and LuxQ, respectively. LuxQ functions in conjunction with the periplasmic binding protein LuxP [8,9]. Two other QS receptors, VpsS and CqsR, have recently been identified but their ligands remain unknown [10,11]. All four QS receptors are hybrid two-component sensor histidine kinases [12–14]. The *V*. *cholerae* system functions as follows (see Fig 1): at LCD, in the absence of autoinducers, CqsS and LuxPQ act as kinases and shuttle phosphate, via the phospho-relay protein LuxU, to the transcription factor called LuxO [15–17]. LuxO~P activates expression of genes encoding a set of small regulatory RNAs called Qrr1-4 [18]. Qrr1-4 post-transcriptionally control the fates of the mRNAs encoding the two QS master transcription factors AphA and HapR. Specifically, the Qrr sRNAs promote production of the LCD master regulator, AphA, and they repress production of the HCD master regulator, HapR [19]. Thus, under this condition, bacteria enact behaviors appropriate for the individual lifestyle. At HCD, CAI-1 and AI-2 bind to and agonize CqsS and LuxPQ, respectively, converting them from kinases to phosphatases. LuxO is dephosphorylated, which inactivates it [20,21]. The Qrr sRNAs are not made, and thus, AphA production is not activated and HapR production is not repressed. HapR controls the expression of genes necessary for group behaviors. Of note, HapR represses expression of genes required for virulence factor production and biofilm formation [9,22–24]. Therefore, strategies that induce the *V*. *cholerae* HCD state render the pathogen avirulent [9,11,25,26]. Indeed, successful synthetic manipulation of *V*. *cholerae* QS through inhibition of LuxO has already been described [27]. Furthermore, commensal *E*. *coli* engineered to produce CAI-1 in the mouse intestine dramatically reduce cholera toxin production, and consequently, the lethality of *V*. *cholerae* infection [28].

LuxS and AI-2 are broadly distributed among bacteria and AI-2 is proposed to function in inter-species communication [4,29]. By contrast, CAI-1 and CqsS are almost exclusively restricted to *vibrios* suggesting they are used for intra-genus communication [3,30]. In *V*. *cholerae*, the CAI-1-CqsS system plays the dominant role relative to the weaker AI-2-LuxPQ system in directing *V*. *cholerae* QS-regulated gene expression, at least under laboratory conditions [9]. Here, we explore natural and synthetic modulation of the *V*. *cholerae* CqsS receptor to probe its overarching role in QS and to explore possibilities for synthetic manipulation of the process. We find that there exist ~40 CqsS dimers per cell at LCD, and QS induces CqsS production, which increases CqsS levels to ~170 dimers per cell during the LCD to HCD transition. By contrast, LuxQ levels remain unchanged as cells transition into QS-mode. The consequence of increased production of CqsS during the QS transition in *V*. *cholerae* is to enhance CAI-1 detection, which, as a result, dampens the response to AI-2. Indeed, phosphatase-active LuxQ receptors are incapable of overriding kinase-active CqsS receptors. Conversely, phosphatase-active CqsS receptors can supersede kinase-active LuxQ receptors. We show that this feature of the network has important implications for the lifecycle of *V*. *cholerae*. Specifically, the *hapA* gene, encoding the dissemination-promoting protease HapA, is highly upregulated in response to CAI-1 but not in response to AI-2. Thus, *V*. *cholerae* is capable of CAI-1-specific responses even though CqsS and LuxPQ signal through a common pathway.

With respect to CqsS function, we identify two classes of synthetic CqsS agonists. Remarkably, although CqsS exhibits exquisite selectivity for CAI-1 when presented with CAI-1 derivatives [31], responding to only a restricted subset of closely related CAI-1 analogs [30], the synthetic compounds we identify, while capable of potently agonizing CqsS, share no structural similarity to CAI-1. The synthetic agonists inhibit CqsS kinase activity, analogous to the mechanism by which CAI-1 functions to control CqsS signal transduction. The synthetic compounds display superior potency and efficacy compared to CAI-1 making them promising leads for *V*. *cholerae* prophylactics and/or therapeutics.

## Results

### CqsS production increases at high cell density in *V*. *cholerae*

**Fig 2:**
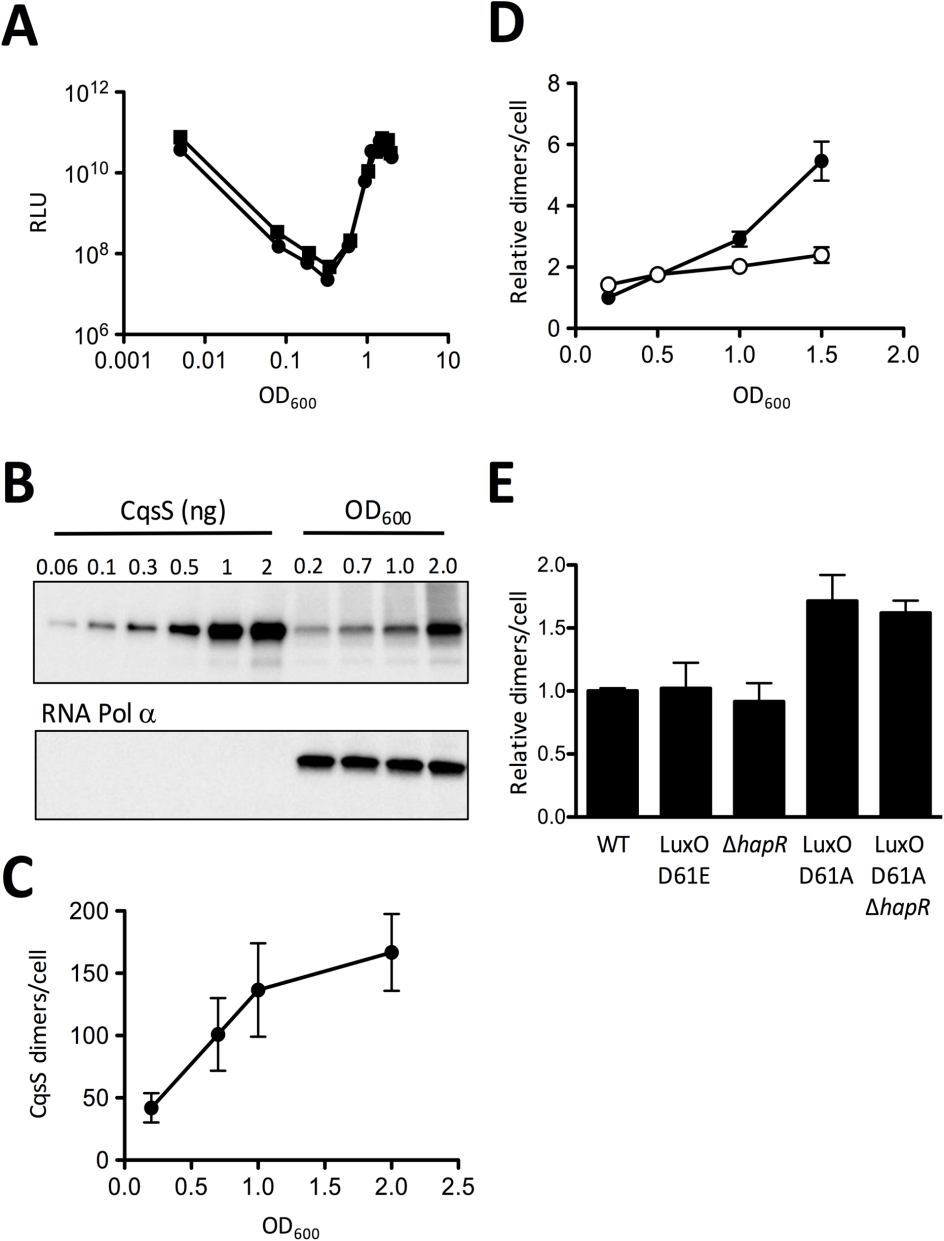
CqsS production increases at high cell density. A) Light production from *V*. *cholerae* carrying WT CqsS (C6706str2, circles) and CqsS::3XFLAG (AH330, squares) and the QS-controlled *luxCDABE* operon. Relative light units (RLU) are defined as counts/min ml^-1^ per OD_600_. The panel shows representative data from n = 3 experiments. B) Quantitative western blot showing the indicated amounts of purified CqsS protein (left) and the amounts of CqsS present in *V*. *cholerae* cells collected at the specified OD_600_ (right). The bottom panel shows the RNA Pol α subunit loading control. Lysate from 0.045 OD_600_ of cells was loaded per well. C) CqsS dimers per *V*. *cholerae* cell at the specified OD_600_ were quantified from panel B by dividing the number of CqsS dimers in each sample by the cell density of the culture, which had been calculated from the colony forming units (CFUs). Error bars represent standard errors of the mean. D) CqsS (closed circles) and LuxQ dimers (open circles) quantified from *V*. *cholerae* strain AH420 (carries CqsS::3XFLAG and LuxQ::3XFLAG) during growth and normalized to the CqsS level at OD_600_ = 0.2, see S2 Fig. Error bars represent standard errors of the mean. E) Relative CqsS dimers per cell in the specified *V*. *cholerae* strains at LCD (OD_600_ = 0.2) normalized to WT levels, see S3A Fig. Error bars represent standard errors of the mean. We note that these errors are smaller than those shown in Fig 2C because they represent relative protein levels rather than absolute protein levels. All experiments were performed three times.

*V*. *cholerae* integrates sensory information from four receptors into a common, downstream QS pathway [9,11]. This network architecture makes it difficult to understand how individual autoinducers could produce unique QS responses. Here, we explore whether feedback operates in *V*. *cholerae* QS which, if so, could provide a possible route to autoinducer-specific responses. We first examined if either or both of the established *V*. *cholerae* receptors is controlled by QS by measuring CqsS and LuxQ levels and activity over growth. We describe in detail the strategy for assessment of CqsS. We used an exactly analogous strategy for analysis of LuxQ. We fused a 3XFLAG epitope to the C-terminus of CqsS and used this construct to replace the native *cqsS* gene on the *V*. *cholerae* chromosome. We verified that the CqsS::3XFLAG fusion functioned properly by monitoring its ability to control the density-dependent expression of luciferase, a heterologous QS reporter (Fig 2A). The *V*. *harveyi luxCDABE* operon is frequently used as a convenient readout of QS-controlled gene expression in *V*. *cholerae*. Indeed, similar to WT *V*. *cholerae*, in the *V*. *cholerae* strain carrying CqsS::3XFLAG, maximal light production occurred following overnight growth (i.e., at HCD). Light output declined precipitously following dilution of the *V*. *cholerae* cells into fresh medium. Dilution reduces the autoinducer concentration to below the threshold required for detection, thereby transitioning the cells into LCD mode. During subsequent growth, autoinducers once again accumulate and are bound by their cognate receptors. Signal transduction activates *lux* expression, and light production commences. The canonical “U” shaped light production curve is the hallmark QS behavior (Fig 2A). Thus, CqsS::3XFLAG functions like WT CqsS. We also deleted the genes encoding the other three QS receptors (Δ*cqsR* Δ*vpsS* Δ*luxQ*) and verified that, like WT CqsS, CqsS::3XFLAG could control gene expression when it was the only QS receptor present (S1A Fig).

We exploited the strain containing the CqsS::3XFLAG construct on the chromosome to examine whether CqsS levels change during QS transitions. Quantitative western blotting (Fig 2B) and the corresponding analysis of the data (Fig 2C) show that CqsS::3XFLAG increases from ~40 dimers per cell at LCD (OD_600_ = 0.2) to ~170 dimers per cell at HCD (OD_600_ = 2.0). We could detect an increase in CqsS::3XFLAG at OD_600_ = 0.7, immediately prior to induction of light production (Fig 2A and 2C) and CqsS levels steadily increased throughout growth. By contrast, similar measurements examining a functional LuxQ::3XFLAG fusion (S1B Fig) show that LuxQ::3XFLAG levels remain relatively unchanged throughout growth (Fig 2D and S2 Fig). Therefore, CqsS production increases during QS whereas LuxQ production does not.

An obvious mechanism that could underpin the increase in CqsS production that occurs with increasing *V*. *cholerae* cell density is regulation of CqsS by QS. To investigate this possibility, we quantified CqsS::3XFLAG levels in *V*. *cholerae* QS mutants that are locked at LCD and HCD. As mentioned above (and see Fig 1), LuxO functions as the QS signal integrator that is phosphorylated and activated at LCD. LuxO D61E is a phosphomimetic allele that constitutively activates expression of the *qrr* genes. Thus, LuxO D61E confers the LCD state irrespective of cell density due to Qrr-mediated activation of *aphA* and repression of *hapR*. Likewise, the Δ*hapR* strain is locked in LCD mode. Conversely, the LuxO D61A allele cannot be phosphorylated so it is inactive and incapable of activating expression of the *qrr* genes. Thus, LuxO D61A confers the constitutive HCD state and HapR is produced at all cell densities. Fig 2E and S3A Fig show that, at LCD, the level of CqsS in the LuxO D61E and the Δ*hapR* strains is equal to that in the WT whereas the LuxO D61A single and the LuxO D61A Δ*hapR* double mutants possess increased CqsS relative to WT and the locked LCD mutants. Thus, CqsS is upregulated in the absence of LuxO~P and in the absence of HapR. This result shows that regulation cannot occur through HapR, but rather, control must be a consequence of LuxO~P-mediated repression at LCD. We deleted *aphA* and there was no effect on CqsS production (S3B and S3C Fig). Thus, regulation must occur via the Qrr sRNAs [32–35]. We note that in the HCD-locked mutants, CqsS levels are upregulated 1.5-fold relative to WT, while CqsS levels in the WT increase ~4-fold as cells transition from LCD to HCD. We suspect that accumulation of autoinducer over time, which occurs in the WT but not in the locked mutants, is required for full regulation of CqsS production.

We examined whether Qrr regulation of CqsS is direct or indirect using a CqsS-mKATE2 translational fusion in *E*. *coli* expressing inducible *qrr*4. We reasoned that, if CqsS production is controlled by Qrr4, repression of CqsS-mKATE2 should occur when Qrr4 is induced. We verified that our system works by showing that inducible Qrr4 is capable of repressing a previously-identified direct target, VCA0107-GFP [24]. However, no repression of CqsS-mKATE2 occurred following induction of Qrr4 (S4 Fig). This result, coupled with the fact that there is no obvious base-pairing region between the Qrr sRNAs and the CqsS mRNA, suggests that Qrr regulation of CqsS is indirect.

### Extracellular CAI-1 levels increase with increasing CqsS levels enabling maximum induction of the *V*. *cholerae* QS response

**Fig 3:**
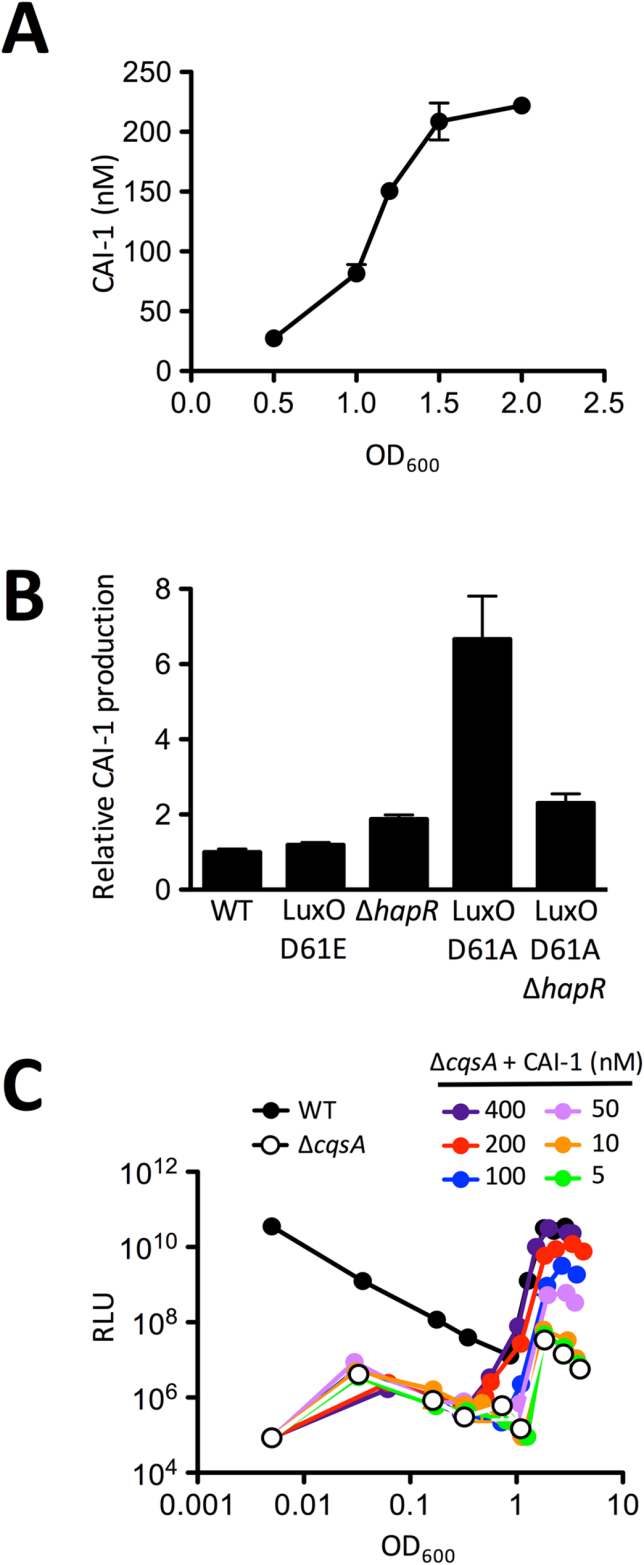
CAI-1 levels dictate the QS response *in vivo*. A) Concentration of CAI-1 in WT *V*. *cholerae* (AH330: carries CqsS::3XFLAG) cell-free culture fluids at the specified OD_600_ calculated from a standard curve using known quantities of synthetic CAI-1 (see S5 Fig). The experiment was performed in triplicate and error bars represent standard errors of the mean. B) CAI-1 production was determined using the *V*. *cholerae* CAI-1 reporter strain WN1102 following administration of 30% cell-free culture fluids prepared from the specified *V*. *cholerae* strains collected at LCD (OD_600_ = 0.2). Values were normalized to the bioluminescence emission induced by WT cell-free culture fluids. The experiment was performed three times and error bars represent standard errors of the mean. Unpaired student’s t test comparing WT to Δ*hapR* and WT to LuxO D61A Δ*hapR* produced p values of 0.0005 and 0.002, respectively. The differences between the WT and the LuxO D61E values were not significant (p = 0.1223). C) Light production from WT *V*. *cholerae (*AH330, black) compared to the *cqsA* mutant (AH370: Δ*cqsA*, carries CqsS::3XFLAG, white is the no-addition control) in response to the indicated amounts of synthetic CAI-1 (multiple colors). Relative light units (RLU) are defined as counts/min ml^-1^ per OD_600_. The assay was repeated three times and the data shown are from a representative experiment.

Our next goal was to define the concentration of CAI-1 that induces the CqsS-directed QS response to understand whether QS regulation of CqsS levels influences CAI-1 detection. It is not possible to directly measure CAI-1-CqsS interactions because, like many multi-pass trans-membrane proteins, CqsS has remained recalcitrant to purification and traditional biochemical analyses [31]. We reasoned that knowing the number of CqsS dimers/cell, the concentration of CAI-1 present in cell-free culture fluids, and the timing of QS induction would enable us to infer the *in vivo* CAI-1:CqsS ratio required for QS activation. Our above data defined the number of CqsS dimers/cell (Fig 2C). We quantified CAI-1 by comparing induction of *V*. *cholerae* QS in response to cell-free culture fluids prepared from WT *V*. *cholerae* to induction in response to known quantities of synthetic CAI-1 added to cell-free culture fluids prepared from a Δ*cqsA* (CAI-1 synthase) mutant (S5 Fig). We could not accurately quantify CAI-1 by mass spectrometry because, as has been reported previously, hydroxyketones are extraordinarily difficult to differentiate from fatty acids in organically-extracted cell-free culture fluids [36]. We assayed the preparations on a *V*. *cholerae* Δ*cqsA* Δ*luxQ* mutant strain carrying luciferase. This strain makes no CAI-1 and it lacks the ability to respond to AI-2. Therefore, it activates light production exclusively in response to exogenously supplied CAI-1. We call this strain the *V*. *cholerae* CAI-1 reporter strain. Using this strategy, we found that the concentration of CAI-1 in *V*. *cholerae* cell-free culture fluids increases from 27 nM at OD_600_ = 0.5 to 220 nM at OD_600_ = 2.0 (Fig 3A). CAI-1 was undetectable in our assay at OD_600_ < 0.5. Together, the CqsS and CAI-1 quantitation allow us to estimate the CqsS:CAI-1 ratios at different cell densities. CqsS levels of ~40 dimers per cell at OD_600_ = 0.2 and ~170 dimers per cell OD_600_ = 2.0 can be converted to 11 pM and 480 pM at those two cell densities, respectively. Therefore, during the transition from LCD to HCD (i.e., above OD_600_ = 0.7), CAI-1 is always in at least a 450-fold molar excess relative to the CqsS receptor.

The *cqsA* gene is located adjacent to but in the opposite orientation of the *cqsS* gene on the *V*. *cholerae* chromosome. Thus, their regulation is not obviously linked. Nonetheless, the increase in CAI-1 over the course of growth could be a consequence of QS control of *cqsA*. To test this possibility, cell-free culture fluids were harvested at LCD from the strains in Fig 2E. Relative CAI-1 concentrations were determined by supplying them to the *V*. *cholerae* CAI-1 reporter strain and comparing bioluminescence output. The WT and the LuxO D61E mutant had the same level of CAI-1 in their culture fluids (Fig 3B). The LuxO D61A mutant produced ~7-fold more. Deletion of *hapR* in the LuxO D61A background diminished induction compared to the single LuxO D61A mutant. Nonetheless, the Δ*hapR* single and the LuxO D61A Δ*hapR* double mutant strain still produced twice as much CAI-1 as the WT suggesting that the increase in CAI-1 cannot stem fully from the loss of LuxO~P mediated repression of *qrr* expression alone. Rather, the results suggest that CAI-1 production is both repressed by LuxO~P at LCD, presumably via the Qrr sRNAs, and activated by HapR at HCD (Fig 3B).

To determine the concentration of CAI-1 required to induce QS *in vivo*, that is, in the presence of a functional LuxPQ pathway, we supplied synthetic CAI-1 to a *V*. *cholerae* Δ*cqsA* strain carrying luciferase. Concentrations of CAI-1 above 200 nM fully induced QS, mimicking what occurs in WT *V*. *cholerae* at HCD in response to endogenously-produced CAI-1 (Fig 3C). Induction of bioluminescence occurred at a CAI-1 concentration of 50 nM, corresponding roughly to the concentration of CAI-1 present in WT *V*. *cholerae* cell-free culture fluids at which the QS response naturally initiates (compare data in Fig 3A and 3C). Concentrations of CAI-1 below 50 nM failed to activate a QS response (Fig 3C). These results agree with the reported 35 nM *K*_*d*_ of CAI-1 for CqsS [31]. We conclude that the CAI-1 ligand and the CqsS receptor, at least under the conditions we tested, exist at levels within the range that ensures high sensitivity to changing CAI-1 concentrations (S6 Fig).

### Upregulation of CqsS production is driven by both CAI-1 and AI-2

**Fig 4:**
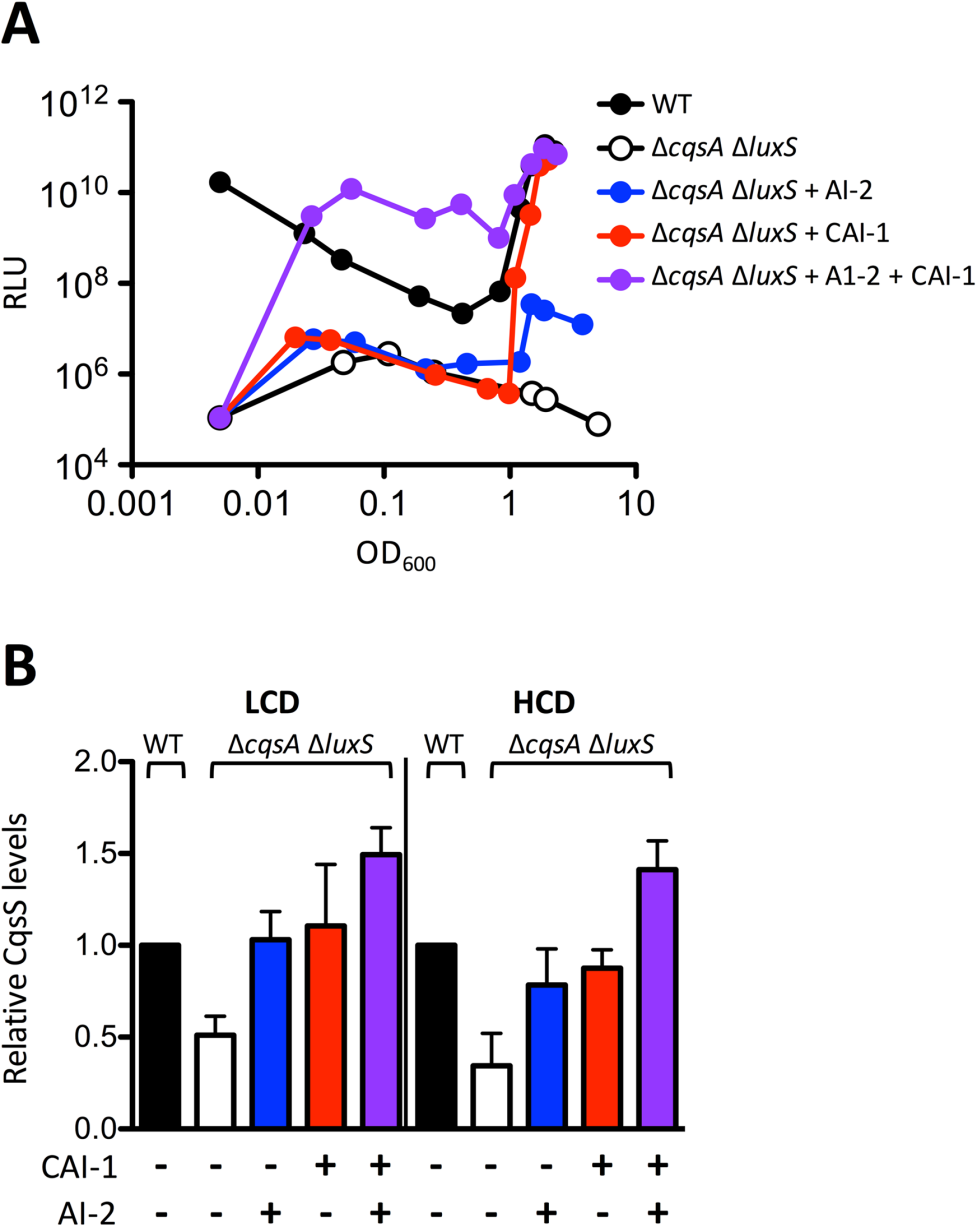
The CAI-1-CqsS system overrides the AI-2-LuxPQ system. A) Bioluminescence output from the QS-controlled *luxCDABE* operon in WT *V*. *cholerae* (AH330: carries CqsS::3XFLAG, black) and the double autoinducer synthase mutant (AH371: Δ*cqsA* Δ*luxS*, carries CqsS::3XFLAG, white) in response to AI-2 (blue), CAI-1 (red), or both AI-2 and CAI-1 (purple) at saturating 1 μM and 5 μM concentrations, respectively. Relative light units (RLU) are defined as counts/min ml^-1^ per OD_600_. The assay was repeated three times and the data shown are from one representative experiment. B) Western blot quantitation of CqsS levels in the Δ*cqsA* Δ*luxS V*. *cholerae* strain AH371 normalized to WT AH330 levels, see S7B Fig. Colors and treatments as in panel A. LCD and HCD conditions are OD_600_ = 0.2 and 2.0, respectively. All experiments were performed in triplicate. Error bars represent standard errors of the mean.

We wondered what consequence feedback on CqsS and CAI-1 has on *V*. *cholerae* QS behavior. To assess this, we measured QS induction in a *V*. *cholerae* Δ*cqsA* Δ*luxS* autoinducer synthase mutant in the presence of one or both autoinducers using bioluminescence as the readout. The double Δ*cqsA* Δ*luxS* autoinducer synthase mutant makes 100,000-fold less light than WT *V*. *cholerae* following overnight growth (Fig 4A, black vs. white symbols and see S7A Fig). Addition of saturating AI-2 increased light production 100-fold at HCD (blue), whereas addition of saturating CAI-1 induced WT levels of light (i.e., 100,000-fold induction; red). Simultaneous addition of both CAI-1 and AI-2 induced light production earlier than did addition of CAI-1 alone (purple). Thus, CAI-1 plays the dominant role in driving QS induction, however, together, the two autoinducers have a synergistic effect. We interpret these results to mean that, in the absence of CAI-1, the phosphatase activity of AI-2-stimulated LuxQ cannot override the kinase activity of the unliganded CqsS receptor. By contrast, in the absence of AI-2, the phosphatase activity of CAI-1-bound CqsS can overcome the kinase activity of the unliganded LuxQ receptor. The presence of LuxQ kinase activity, however, delays induction of the QS response to CAI-1 when it is the only autoinducer present.

Quantitation of CqsS levels in the above strains shows that the amount of CqsS protein in the Δ*cqsA* Δ*luxS* mutant was significantly lower than that in WT at both LCD and HCD (Fig 4B). Addition of either AI-2 (blue) or CAI-1 (red) increased CqsS protein levels to nearly that present in the WT at LCD and HCD. Simultaneous administration of both CAI-1 and AI-2 (purple) increased the CqsS levels to above WT levels, again both at LCD and HCD. These data show that autoinducers control CqsS production via feedback and they act synergistically to do so. Moreover, these data suggest that, even at LCD, *V*. *cholerae* can detect the presence of either single autoinducer, and as a consequence, via feedback, increase CqsS production.

The feedback-driven increase in CqsS production that occurs at HCD should confer an increase in CqsS-mediated dephosphorylation of LuxO~P relative to that mediated by LuxQ. To examine the relative phosphatase activities of the QS receptors, we measured expression of *qrr*4, the direct target of LuxO~P (S8 Fig). For this analysis, we employed a *qrr*4-*luxCDABE* transcriptional fusion that is activated by LuxO~P at LCD. *qrr*4-*luxCDABE* expression was 50-fold higher in the locked LCD Δ*cqsA* Δ*luxS* double autoinducer synthase mutant than in WT *V*. *cholerae* because LuxO is maximally phosphorylated in the double synthase mutant (both CqsS and LuxQ are kinases) and maximally dephosphorylated in the WT (both CqsS and LuxQ are phosphatases). Addition of saturating AI-2 and addition of saturating CAI-1 to the Δ*cqsA* Δ*luxS* double autoinducer synthase mutant reduced *qrr*4-*luxCDABE* expression 2-fold and 20-fold, respectively. Importantly, CAI-1 had a 10-fold greater effect than did AI-2 showing that CqsS, the receptor that experiences positive feedback, has the stronger phosphatase activity. Addition of both autoinducers together further repressed *qrr*4-*luxCDABE* expression to WT levels, demonstrating the synergy between the two ligands (S8 Fig).

We suggest that CqsS exhibits stronger phosphatase activity on LuxO~P than does LuxQ because QS-mediated positive feedback increases CqsS levels, making it the dominant receptor. This hypothesis predicts that altering receptor ratios could transfer dominance to LuxQ. To explore this prediction, we measured the QS output when chromosomal *cqsS* was placed under the control of the *luxPQ* promoter. This arrangement reduced CqsS production by 80% compared to when *cqsS* was expressed from its native promoter (S9A Fig). Under this condition, AI-2 caused premature induction of QS, and the AI-2 input-output range was expanded relative to the AI-2 response shown in Fig 4B (S9B Fig). By contrast, CAI-1 was incapable of inducing the premature response (S9B Fig). Together, these results highlight the importance of receptor ratios in the proper control of QS behavior. Furthermore, the data show that, in WT *V*. *cholerae*, positive feedback on CqsS dampens the response to AI-2.

### The CAI-1-CqsS pathway controls expression of genes that are not controlled by the AI-2-LuxPQ pathway

**Fig 5:**
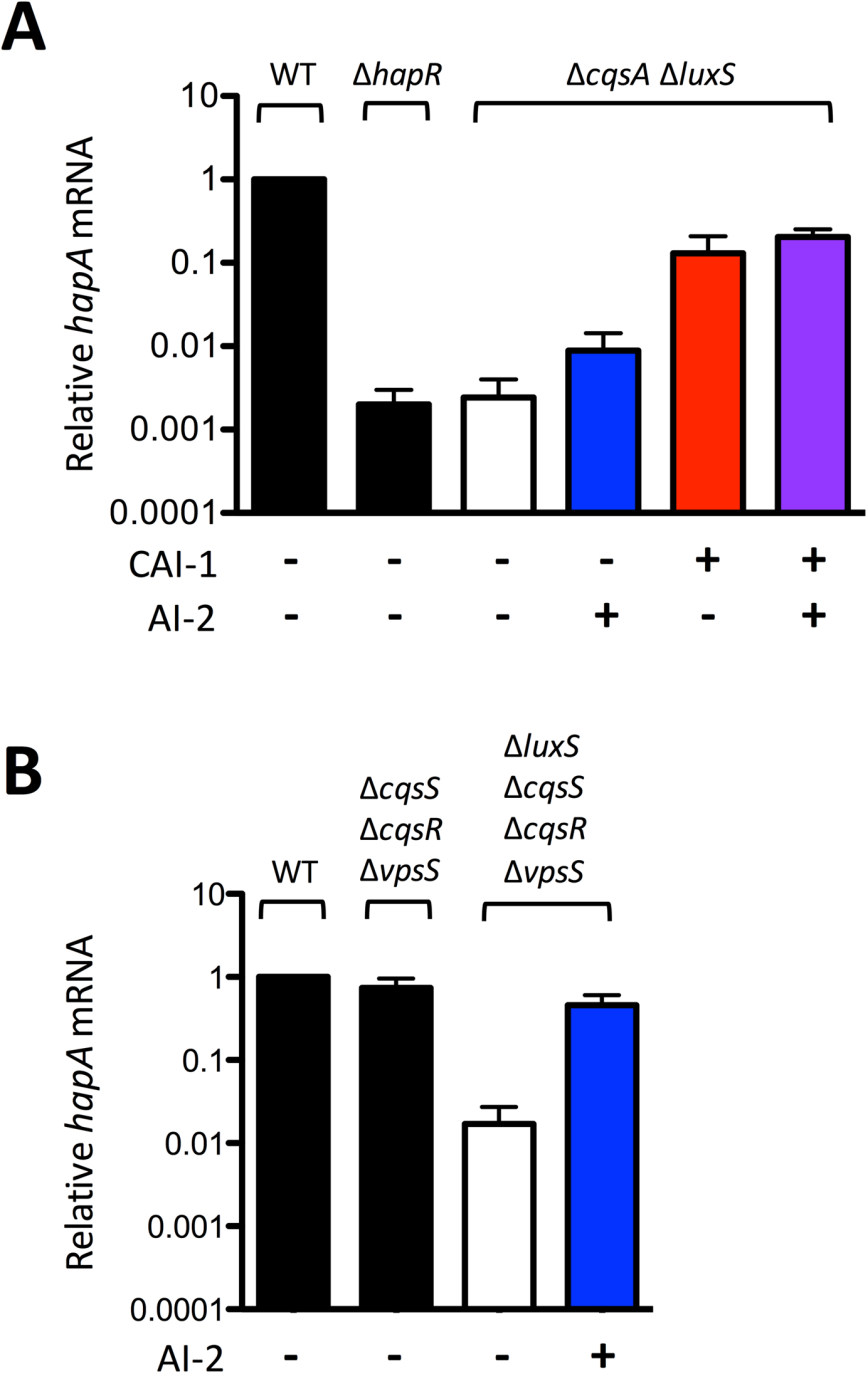
The CAI-1-CqsS pathway controls specific genes. qRT-PCR of *hapA* at HCD (OD_600_ = 2.0) in: A) WT (C6706str2), Δ*hapR* (MM239), and Δ*cqsA* Δ*luxS* (MM914) *V*. *cholerae* strains and B) WT, LuxQ^+^AI-2^+^ (WN3627: Δ*cqsS* Δ*vpsS* Δ*cqsR*) and LuxQ^+^AI-2^-^ (AH464: Δ*cqsS* Δ*vpsS* Δ*cqsR* Δ*luxS*) strains. AI-2 and CAI-1 were provided at 1 μM and 5 μM, respectively. Means and standard errors of the mean for three independent experiments are shown. Transcript levels were normalized to *hfq* mRNA.

The differential QS induction that *V*. *cholerae* exhibits in response to CAI-1 versus AI-2 (see Fig 4A) stems from the upregulation of the CAI-1-CqsS pathway that occurs during the QS transition. Specifically, at LCD, there are roughly equal numbers of CqsS and LuxQ dimers (CqsS:LuxQ = 0.7:1). Following the transition to HCD, the CqsS:LuxQ ratio increases to 2.5:1 (Fig 2D). We have shown that feedback onto CqsS enables it to disproportionately dephosphorylate LuxO~P (S8 Fig), which, consequently, must increase HapR levels. If so, QS-controlled genes could be regulated exclusively by the CAI-1-CqsS pathway. To investigate this notion, we measured expression of the QS-activated gene *hapA* [22,37]. *hapA* encodes a protease that is reported to be crucial for *V*. *cholerae* dissemination following infection [38,39]. We used qRT-PCR to measure *hapA* transcript in HCD cultures of WT *V*. *cholerae*, the Δ*hapR* mutant, and the Δ*cqsA* Δ*luxS* double autoinducer synthase mutant (Fig 5A). HapR activates *hapA* expression, and consistent with this, the *hapA* transcript was nearly undetectable in the Δ*hapR* strain (black). Likewise, the locked LCD double autoinducer synthase (Δ*cqsA* Δ*luxS*, white) mutant in which HapR is not made (see Fig 1) produced levels of *hapA* transcript similar to the Δ*hapR* mutant. Addition of AI-2 to the Δ*cqsA* Δ*luxS* strain increased *hapA* expression 4-fold (blue) while addition of CAI-1 increased *hapA* transcript levels over 50-fold (red). Simultaneous addition of both autoinducers (purple) increased *hapA* expression an additional 2-fold above that following addition of CAI-1 alone.

The above experiment, together with the results in S9B Fig, suggests that the stoichiometry of QS receptors dictates the level of *hapA* expression. To verify this hypothesis, we exchanged QS control of *hapA* from CqsS to LuxQ by deleting the genes encoding all of the QS receptors except LuxQ (Δ*cqsS* Δ*cqsR* Δ*vpsS*). We quantified the levels of *hapA* in this strain, which we call the LuxQ^+^ AI-2^+^ strain and compared them to a LuxQ^+^ AI-2^-^ strain (Δ*cqsS* Δ*cqsR* Δ*vpsS* Δ*luxS*) that possesses LuxQ but cannot make AI-2 (Fig 5B). The LuxQ^+^ AI-2^-^ strain had 44-fold less *hapA* transcript than the LuxQ^+^ AI-2^+^ strain. Addition of AI-2 to LuxQ^+^ AI-2^-^ strain increased *hapA* expression 27-fold. Thus, AI-2 can control *hapA*, but only in the absence of CqsS. We have used *hapA* as our representative test case. We reason that other QS-controlled genes behave similarly [9,40–44]. This result suggests that QS control of receptor ratios dictates discrete gene expression patterns in *V*. *cholerae* at HCD, and the downstream effect is dominated by CAI-1-CqsS.

### Synthetic compounds that are not structurally related to CAI-1 can agonize *V*. *cholerae* CqsS

**Fig 6:**
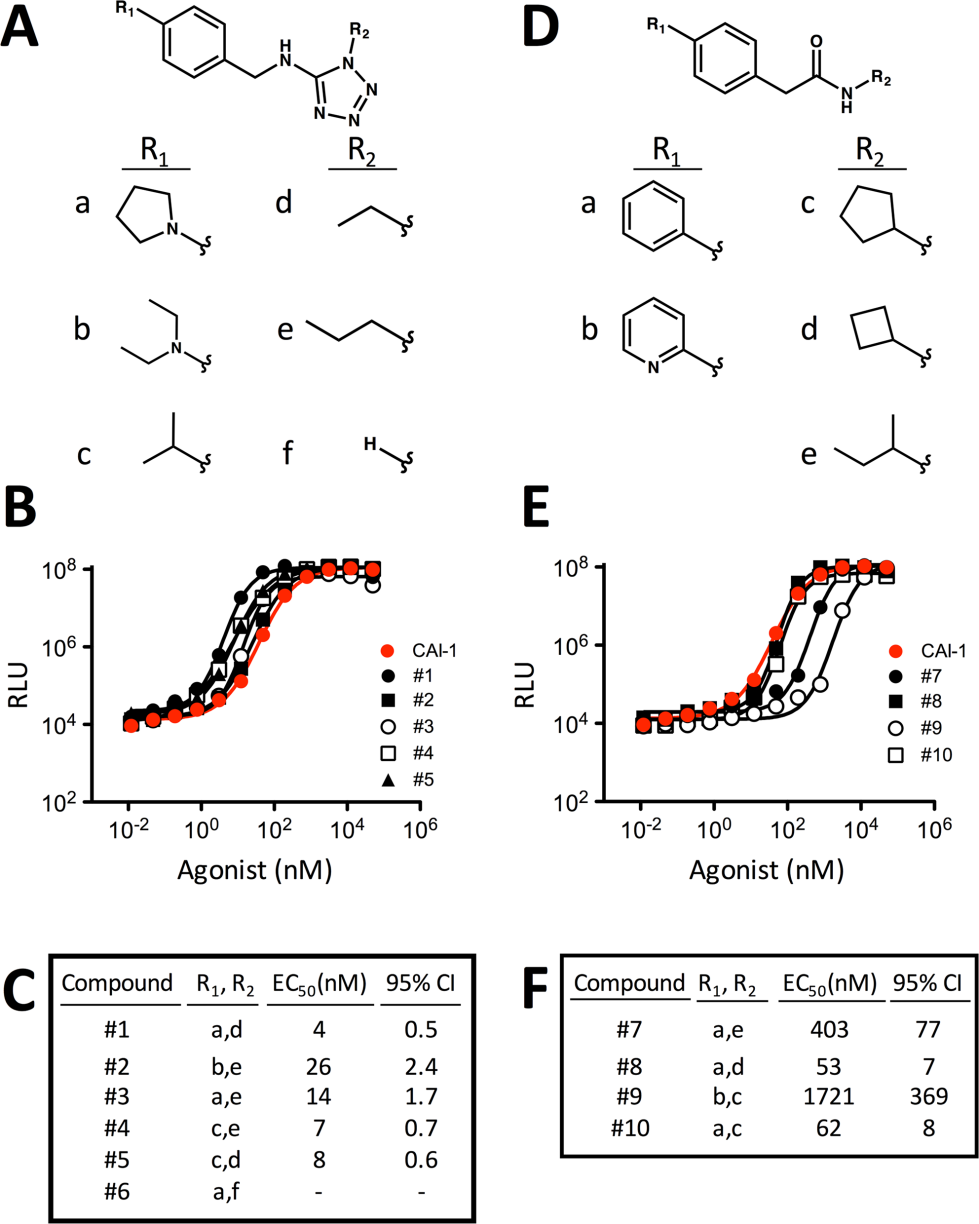
Structures and activities of synthetic CqsS agonists. Structures (A and D), activities (B and E), EC_50_ values and 95% confidence intervals (CI) (C and F) of Class 1A and Class 1B CqsS synthetic agonists, respectively. In panels B and E, light-production from the *V*. *cholerae* CAI-1 reporter strain WN1102 is shown in response to increasing concentrations of CAI-1 (red) and the designated synthetic compounds. The reported EC_50_ for CAI-1 is 35 nM [31], we measure it here to be 38 nM. Relative light units (RLU) are defined as counts/min ml^-1^ per OD_600_. All experiments were performed in triplicate. Error bars showing standard errors of the mean are present, albeit small, in panels B and E.

The above findings suggest that agonizing the major QS receptor CqsS could influence *V*. *cholerae* dispersal, making CqsS an excellent target for small molecule manipulation. *V*. *cholerae* CqsS responds to the cognate CAI-1 ligand (*S*)-3-hydroxytridecan-4-one and the close analogs enamino-CAI-1 (Z)-3-aminotridecan-2-en-4-one and enamino-C8-CAI-1, made by related *vibrios* [30]. However, CqsS does not activate QS in response to CAI-1 analogs with enlarged head groups or shortened acyl tails [31]. We probed the limits of CqsS ligand detection by assessing a library of synthetic compounds for CqsS agonism. We screened 352,083 compounds at 20 μM for those that induced bioluminescence 10,000-fold in the double synthase mutant (Δ*cqsA* Δ*luxS*) carrying luciferase. Putative hits were re-tested at a variety of concentrations and active compounds were selected. We employed a secondary screen using the Δ*cqsS* mutant carrying luciferase to identify the subset of active compounds that require CqsS for function [27]. Nine compounds were identified that met these criteria. They were further subdivided into two groups (denoted 1A and 1B) based on their structures (Fig 6A and 6D). None of the compounds share structural similarity with CAI-1.

Class 1A compounds are 5-aminotetrazole derivatives and all are more potent than CAI-1 (EC_50_ = 38 nM; Fig 6B) and all are equally efficacious as CAI-1 at agonizing CqsS (Fig 6A–6C). The most potent compound in this class, compound #1, is ~10-fold more potent than CAI-1 with an EC_50_ = 4 nM (Fig 6C). Compound #3, in which the R2 ethyl group of compound #1 is replaced by the longer propyl side chain, is approximately 4-fold less potent. Compound #6, containing a free N-H at R2, loses all activity (S10 Fig), indicating an absolute requirement for a substituent at the R2 position. This result could be due to either stereoelectronic or conformational considerations; the limited dataset does not allow a deeper explanation. The R1 substituent has a minor effect on potency. The N,N-diethylanilino moiety appears to slightly decrease potency relative to the pyrrolodino moiety (compound #2 vs. compound #3) while the isopropyl group shows both a modest increase (compound #4 vs. compound #3) and a slight reduction (compound #5 vs. compound #1) in activity relative to the pyrrolodino moiety, depending on the nature of the R2 substituent. These results suggest that the aniline nitrogen present in compounds #1–3 is not crucial for activity. Class 1B molecules are biphenyl amide derivatives, and all are less potent than CAI-1 (Fig 6D–6F). This initial dataset suggests that cyclic R2 substituents on the secondary amide are preferred over acyclic ones (compare compounds #8 and #10 vs. compound #7). In addition, substitution of a 2-pyridine ring in place of the benzene ring at position R1 (compound #9) significantly diminished potency.

### Synthetic CqsS agonists function by inhibiting *V*. *cholerae* CqsS kinase activity

**Fig 7:**
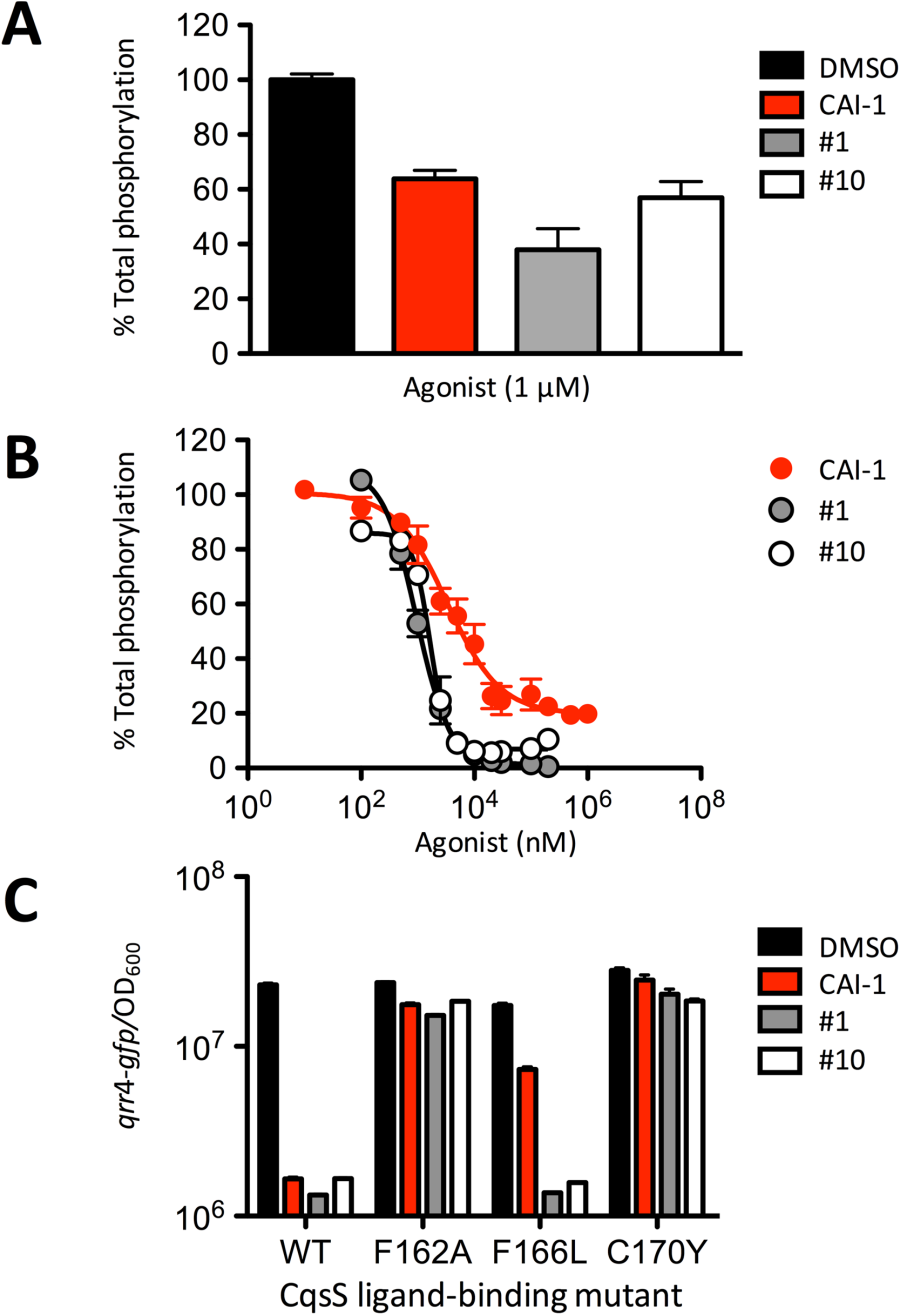
Synthetic agonists inhibit CqsS autophosphorylation. A) Inhibition of autophosphorylation of CqsS D618N by 1 μM CAI-1 (red), Class 1A compound #1 (gray), and Class 1B compound #10 (white). Reactions were subjected to gel electrophoresis and each gel contained an independent DMSO control. Gels from a single experiment were exposed simultaneously. Band intensities for CqsS D618N~P were normalized to the maximum phosphorylation in presence of DMSO (black), see S11A Fig. Experiments were performed in triplicate and error bars represent standard errors of the mean. B) Concentration-dependent inhibition of WT CqsS autophosphorylation by CAI-1 and representative synthetic agonists, see S11B Fig. Experiments were performed in duplicate and error bars represent standard errors of the mean. C) CqsS-dependent repression of *qrr*4*-gfp* expression following addition of DMSO, 1 μM CAI-1, compound #1, or compound #10 when WT CqsS or the designated CqsS variant is present in *V*. *cholerae*. Experiments were performed in triplicate and error bars represent standard errors of the mean. We note that slightly different EC_50_/IC_50_ values are obtained in the two assays used in this figure and in the bioluminescence assay used in Fig 6B and 6E. Different EC_50_ values are obtained because the QS components measured in the assays function in different tiers of the cascade and are related in a non-linear fashion.

CAI-1 inhibits CqsS autokinase activity, whereas CqsS phosphatase activity is not altered by CAI-1 binding [17]. Thus, in the unliganded state, CqsS kinase prevails, and in the CAI-1-bound state, CqsS phosphatase activity dominates. We tested whether the newly identified synthetic agonists functioned by the same mechanism. CqsS autophosphorylation can be assayed using inverted membrane vesicles containing CqsS protein and measurements of incorporation of ^32^P. Using a CqsS mutant that cannot transfer the phosphate to the receiver domain (CqsS D618N), we can test the ability of the compounds to specifically inhibit CqsS H194 phosphorylation. Similar to CAI-1, all of the synthetic agonists inhibited the first biochemical reaction in signal relay: CqsS autophosphorylation (Fig 7A shows two representative examples, one from Class 1A and one from Class 1B). Both classes of synthetic agonists, irrespective of whether their EC_50_’s are higher or lower than that of CAI-1, contain compounds with higher efficacy than CAI-1. Specifically, Fig 7B shows that saturating CAI-1 inhibited 80% of WT CqsS autophosphorylation while compound #10 inhibited 90% and compound #1 inhibited 99% of the activity.

The finding that the synthetic compounds function by the same mechanism as CAI-1 to control CqsS signal transduction was surprising given their varied structures relative to the CAI-1 ligand. We therefore examined if the synthetic compounds required the putative CAI-1 binding site for activity. The CqsS F162A, CqsS F166L, and CqsS C170Y variants do not respond to CAI-1 [31]. These amino acid residues are presumed to directly interact with the CAI-1 ligand because changes in the CAI-1 structure that render it inactive can be compensated by companion changes in these key amino acid residues. Specifically, if a bulky moiety is exchanged for a small entity on the CAI-1 ligand, replacing a small amino acid with a bulky one in the CqsS ligand-binding site restores activity, and vice versa. This previous chemical-genetics analysis led to the understanding that in CqsS, F162 and F166 specify the CAI-1 head group and C170 determines the CAI-1 tail length [31]. We used this set of CqsS mutants to explore the requirements for synthetic agonist activity. To do this, we assayed CqsS-directed repression of Qrr4-GFP production. As a reminder, at LCD, when CqsS is unliganded, phospho-relay to LuxO activates Qrr production. At HCD, CAI-1 binding to CqsS terminates Qrr production (see Fig 1 and S8 Fig). Thus, reduced Qrr4-GFP production corresponds to CqsS agonism via inhibition of autokinase activity.

CqsS F162A and CqsS C170Y could not be agonized by either class of synthetic compounds discovered here: Qrr4-GFP production remained constant and at high levels even when the compounds were provided at 1 μM (Fig 7C). Therefore, these residues are crucial for synthetic compound detection. Conversely, CqsS F166L, while nearly impervious to CAI-1, repressed Qrr4-GFP production in response to both Class 1A and 1B compounds, suggesting that F166 is dispensable for signaling by these particular synthetic compounds (Fig 7C). We conclude that the synthetic agonists suppress CqsS kinase activity by employing the native ligand-binding site. The synthetic agonists may make other interactions with CqsS, distinct from those made by CAI-1, that enable potent binding while eliminating the requirement for the F166 residue.

## Discussion

*V*. *cholerae* integrates the information contained in the intra-genus autoinducer CAI-1 and the inter-species autoinducer AI-2 into a shared QS pathway via modulation of the kinase:phosphatase activities of the CqsS and LuxQ receptors, respectively [9]. Deducing the ratio of CAI-1:AI-2 could inform *V*. *cholerae* about whether it is the majority or minority species in the environment. We presume that it would be beneficial for *V*. *cholerae* to exhibit distinct behaviors depending on whether or not it predominates in any particular consortium. Here, we show that a QS-mediated positive feedback loop promotes increased production of CqsS, but not LuxQ during the QS transition from LCD to HCD. CqsS increases from ~40 dimers per cell at LCD (OD_600_ = 0.2) to ~170 dimers per cell at HCD (OD_600_ = 2.0), which translates to a change from 11 pM to 480 pM in the culture. We find that CAI-1 production is similarly upregulated, with levels increasing from 27 nM to 220 nM from LCD (OD_600_ = 0.5) to HCD (OD_600_ = 2.0). Concentrations of CAI-1 that highly exceed those of the CqsS receptor during the transition from LCD to HCD make the increase in CqsS levels inconsequential with respect to the ability of the receptor to detect CAI-1. A previous study examining yeast strains containing different so-called secrete-and-sense circuits concluded that cells exhibiting high receptor levels relative to ligand levels were “asocial” because they capture their own ligand, which prevents neighboring cells from detecting released ligand [45]. By contrast, cells with low receptor levels coupled with high ligand secretion rates favored social communication by preventing “self-communication.” Such logic could apply to the *V*. *cholerae* QS CqsS receptor.

Our results suggest that the crucial consequence of positive feedback on CqsS is to alter the potency of CAI-1-driven intra-cellular signaling relative to that driven by AI-2, which enables CAI-1-specific QS outputs. Similar to what we show here for *hapA*, CAI-1-specific regulation in *V*. *cholerae* has been reported previously for the heterologous bioluminescence readout, for biofilm formation, and for chemotaxis [9,43,44]. Here, we have defined the mechanism: asymmetric positive feedback onto CqsS underlies these observations. This feature of the QS architecture could be applicable to other genes in the QS pathway and to other signal transduction systems responsive to multiple inputs in which information is funneled internally through parallel pathways.

Receptor ratio modulation should have specific consequences for how effectively *V*. *cholerae* monitors its cell density in mono-culture and in mixed-species communities. We take the case of mono-culture first: at LCD, *V*. *cholerae* possesses low, but approximately equal numbers of CqsS and LuxQ dimers which fosters roughly equal sensitivity to the CAI-1 and AI-2 signal inputs. Under this condition, exogenous addition of either single autoinducer fails to prematurely induce any QS output whereas simultaneous addition of CAI-1 and AI-2 launches the QS response (Fig 4A). We attribute the lack of response to either individual autoinducer to the overriding kinase activity of the remaining, unliganded receptor. Such an arrangement could protect the system from transient fluctuations in a single autoinducer. Insulation of the QS response from premature activation due to information flowing through a single channel has been reported previously [11,46] and could be a conserved attribute of *vibrio* QS circuits, which, as far as is known, all have similar network architectures. Consistent with this idea, we found that overexpression of *cqsS* in a Δ*cqsA* strain resulted in a severe growth defect when exogenous CAI-1 was supplied. We suspect that it is crucial to maintain low CqsS levels at LCD because the mis-regulation of a downstream QS target(s) under this condition is lethal. We are currently investigating the mechanism underpinning the LCD growth defect.

With respect to receptor ratios at HCD in *V*. *cholerae* mono-cultures, here we find that a mechanism exists to ensure an asymmetric increase in production of CqsS relative to LuxQ during the transition into QS mode. Previously, receptor ratio modulation was studied in the related bacterium, *V*. *harveyi*. *V*. *harveyi* employs three autoinducer-receptor pairs and possesses a feedback loop controlling receptor levels for the AI-1-LuxN pathway, a pathway that does not exist in *V*. *cholerae* [47,48]. Positive feedback on LuxN increased the AI-1 input range, allowing *V*. *harveyi* to enhance its sensitivity to AI-1 relative to AI-2 (and presumably CAI-1, but that was not explicitly tested) at HCD [49,50]. Our results support this notion: positive feedback on CqsS in *V*. *cholerae* likewise diminishes the relative sensitivity to AI-2 at HCD. Here we find that this feature of the network endows *V*. *cholerae* with the ability to control genes exclusively in response to one particular autoinducer, CAI-1, and indeed, CAI-1 alone is capable of fully launching the *V*. *cholerae* QS response (Figs 4 and 5). This finding differs fundamentally from what was shown in *V*. *harveyi*: the *V*. *harveyi* QS response cannot be fully induced unless all autoinducers are present [40,46]. We know that, in the absence of the CqsS receptor, AI-2 signaling through LuxQ can fully induce the *V*. *cholerae* QS response (Fig 5B). Thus, the asymmetry in CAI-1 response that we discovered stems exclusively from positive feedback on CqsS which changes the stoichiometry of the two major QS receptors [49–53]. Biasing the system to favor the intra-genus CAI-1 autoinducer at HCD could be a type of kin-discrimination, ensuring that expensive public goods are not produced until sufficient numbers of kin are present [54].

Now we consider the consequences of feedback on CqsS for *V*. *cholerae* in multi-species consortia. The *V*. *cholerae* lifecycle involves transitions between the human host and the aquatic environment [55–57]. We first consider the case in the environment. If *V*. *cholerae* is at LCD and other *vibrios* are present, *V*. *cholerae* should rapidly launch its QS cascade because other *vibrios* produce AI-2 and different CAI-1 moieties. *V*. *cholerae* CqsS responds to CAI-1, enamino-CAI-1 (Ea-CAI-1), and Ea-C8-CAI-1. Common marine *vibrio* species produce these molecules [30]. Thus, if other *vibrio* species are present, both autoinducer signals could be present, which is the necessary condition to enable rapid activation of the *V*. *cholerae* QS system. Presumably, *vibrios* that use the same autoinducer and share a niche could more successfully colonize habitats via coordinated production of public goods. We predict that this feature could be useful in enabling *V*. *cholerae*, together with its close *vibrio* relatives, to efficiently colonize marine hosts such as copepods, algae, and fish [56,58–61].

We consider the scenario in which *V*. *cholerae* enters a multi-species environment devoid of *vibrios*: the human host. In this case, we expect AI-2 to be the predominant signal encountered since AI-2 is broadly made by bacteria [29,62–64]. *V*. *cholerae* should not react to AI-2 because, as we have shown, at LCD, the *V*. *cholerae* QS system is impervious to any single autoinducer. Under this condition, as *V*. *cholerae* grows within the consortium, the CAI-1 concentration should track with increasing *V*. *cholerae* cell density since, presumably, only *V*. *cholerae* is contributing CAI-1 to the human host milieu. By contrast, AI-2 should accumulate disproportionately relative to *V*. *cholerae* cell numbers. As *V*. *cholerae* reaches HCD, biasing the response to CAI-1 via feedback on CqsS would inflate the ability of *V*. *cholerae* to detect numbers of its kin within a mixed-species community. Indeed, feedback on the *V*. *harveyi* AI-1-LuxN pathway was proposed to allow *V*. *harveyi* to “pay more attention” to AI-2 at LCD and to AI-1 at HCD [49,50]. Our results with *V*. *cholerae* are similar with respect to CAI-1 (Fig 4A).

Now we put the above results into the context of infection. *V*. *cholerae* presumably enters the host in the LCD QS state and so it is in biofilm-formation-mode and actively producing virulence factors [9,22,43] (see Fig 1). We presume this genetic program primes *V*. *cholerae* for successful infection. Upon entrance into the small intestine, *V*. *cholerae* likely encounters a bolus dose of AI-2 but that does not affect biofilm formation or virulence factor expression since both CAI-1 and AI-2 are required at LCD to alter the QS response. We suggest that this arrangement allows *V*. *cholerae* to delay launching its dispersal program until it has grown to significant cell numbers in the host [65]. Positive feedback onto CqsS, however, ensures that once launched, *V*. *cholerae* remains committed to the dispersal program by exaggerating its response to CAI-1 irrespective of whether AI-2 levels are high or low or fluctuating. We do note that high AI-2 levels would intensify the expression of the *V*. *cholerae* HCD program. Importantly, accumulation of the threshold CAI-1 concentration required for the QS response would be the key event that signifies the completion of the infection cycle since CAI-1 alone is sufficient to cause a QS response while AI-2 cannot trip the system in the absence of CAI-1.

The CqsS-dominated QS system of *V*. *cholerae* makes CqsS a possible target for small molecule manipulation. Here, we have identified two classes of CqsS synthetic agonists. While they are structurally dissimilar from the native CAI-1 autoinducer, they inhibit CqsS H194 autophosphorylation similarly to CAI-1. Interestingly, some of the synthetic agonists can inhibit CqsS autophosphorylation more effectively than can the native CAI-1 autoinducer. Conceivably, such a CqsS agonist could more potently and more prematurely induce QS than does CAI-1 resulting in the execution of the *V*. *cholerae* host-dispersal program. Thus, small molecule CqsS agonists could be envisioned as treatments or preventatives for cholera disease, especially in the presence of microbiota-produced AI-2.

Collectively, the results presented in this work support the growing hypothesis that bacteria have mechanisms to uncouple some behaviors from the bulk of the QS-controlled gene expression program, and in so doing, uncouple a portion of the QS response from the constraint of strict cell-density dependence. *Pseudomonas aeruginosa* uses two autoinducers with distinct decay rates to accomplish this feat [66]. The alternative VqmR/VqmA QS pathway in *V*. *cholerae* responds to a microbiota-produced cue called DPO to control biofilm production independently of the genes controlled by the canonical QS signal transduction pathway discussed here [67]. In addition, receptor ratio regulation could provide a mechanism for *V*. *cholerae* to distinguish when it is a majority species in a consortium and, in response, to control a subset of QS target genes. We expect that such information-processing features embedded in larger QS circuits provide plasticity to the systems enabling bacteria to more successfully and appropriately orchestrate group behaviors.

## Materials and methods

### Bacterial strains, culture conditions, and chemical methods

All *V*. *cholerae* strains are derived from wild-type C6706str2 [68]. *E*. *coli* S17-1 λ*pir* was used for cloning and *E*. *coli* C43 (DE3) was used for protein overexpression. *V*. *cholerae* and *E*. *coli* were grown in LB medium at 37^°^C with shaking. Bioluminescence assays were conducted in SOC medium supplemented with tetracycline. Antibiotic concentrations used are as follows: ampicillin, 100 mg/L; kanamycin, 100 mg/L; chloramphenicol, 10 mg/L; tetracycline, 10 mg/L; streptomycin, 5 g/L; polymixin B, 50 U/L. Chemical syntheses of CAI-1 and AI-2 have been described [3,31,69,70]. To identify CqsS agonists, the Broad Institute’s 352,083 compound library was screened as described [27,71].

### DNA manipulation and mutant construction

Alterations to the *V*. *cholerae* genome were generated using the pKAS32 allelic exchange method [72]. Construction of 3XFLAG fusions on pKAS32 plasmids was accomplished via splicing by overlap extension at the C-termini. In all strains carrying CqsS::3XFLAG in which *cqsA* was also mutated (AH366, AH367, AH370, AH371, and AH468), the *cqsS* gene was first fused to *3XFLAG* on the chromosome. *cqsA* was next disrupted by deleting a T (TTT to AA-) in codon 9, the consequence of which, was introduction of a stop codon at codon 14. These strains are used in Fig 3, Fig 4, S7 Fig and S9 Fig. In those figure panels, for simplicity, we call the *cqsA* mutation Δ*cqsA*. The CqsS::3XFLAG overexpression vector was constructed by overlap extension PCR from the pET21b plasmid containing the gene encoding CqsS::6XHis [17]. The LuxQ::3XFLAG overexpression vector was constructed by amplifying *luxQ* from the genome of a *V*. *cholerae* strain (AH420) carrying *luxQ*::*3XFLAG* by exploiting flanking 5’ NcoI and 3’ BamHI sites. The insert was ligated into similarly digested pET21b. The NcoI site introduced a mutation (D2N) into the gene. It was corrected with using Quikchange II XL Site-Directed Mutagenesis (Stratagene). PCR reactions used iProof DNA polymerase (Bio-Rad).

### Quorum-sensing assays

To quantify the function of the CqsS::3XFLAG construct in *V*. *cholerae*, overnight cultures of *V*. *cholerae* strains carrying *luxCDABE* were diluted to OD_600_ = 0.005 in fresh medium and grown at 30^°^C with shaking. Every hour, bioluminescence and OD_600_ were measured on a Tri-Carb 2810 TR scintillation counter and DU800 spectrophotometer, respectively. Samples with OD_600_ > 0.7 were back-diluted 10-fold prior to analysis to ensure measurements could be made within the linear range of the spectrophotometer. CAI-1 was dissolved in DMSO and AI-2 was dissolved in water. The autoinducers were added at the indicated concentrations at the time of dilution. DMSO was used as the negative control and 100 μM of boric acid was added to cultures containing AI-2.

For relative CAI-1 activity assessment, an overnight culture of the *V*. *cholerae* CAI-1 reporter strain (WN1102: Δ*cqsA*, Δ*luxQ*/pBB1) was diluted 1:10 into sterile medium and 30% (v/v) cell-free culture fluids prepared from the indicated *V*. *cholerae* strains were added to the diluted reporter strain. The plasmid pBB1 carries the *V*. *harvyei luxCDABE* genes. Bioluminescence and OD_600_ were measured after the cultures were incubated at 30^°^C for 2.5 h with shaking. To quantify absolute CAI-1 concentration, the bioluminescence from the CAI-1 reporter strain supplemented with 30% cell-free culture fluids from WT *V*. *cholerae* was compared to the bioluminescence from the reporter strain supplemented with known concentrations of synthetic CAI-1 in 30% cell-free Δ*cqsA V*. *cholerae* cell-free culture fluids. DMSO was added to the cell-free WT culture fluids as a negative control. The concentration of endogenously produced CAI-1 in the cell-free culture fluids was extrapolated from the log (agonist) vs. response variable slope calculation for the synthetic CAI-1-directed bioluminescence output using Prism software.

The relative phosphatase activities of the QS receptors were measured in WT *V*. *cholerae* and in the Δ*cqsA* Δ*luxS* double autoinducer synthase mutant using a *qrr*4-*luxCDABE* transcriptional fusion. Overnight cultures were diluted 1:20 in fresh medium in the presence and absence of exogenous autoinducers and incubated 4 h at 30^°^C with shaking. Bioluminescence was measured on a Tri-Carb 2810 TR scintillation counter. Bioluminescence was normalized to OD_600_, which was measured on a DU800 spectrophotometer.

To determine the EC_50_ of synthetic CqsS agonists, the CAI-1 reporter strain WN1102 was diluted 1:20 in fresh medium. The indicated concentrations of CAI-1 or synthetic agonist were added in triplicate to 96-well plates and incubated for 4 h at 30^°^C with shaking. Bioluminescence and OD_600_ were measured on an Envision 2103 Multilabel Reader (Perkin Elmer). We note that the baseline for bioluminescence is lower on the plate reader compared to that from the scintillation counter. We use relative light units rather than absolute light output, which circumvents issues arising from the different sensitivities of the instruments. The EC_50_ of each compound was calculated using Prism software. To assess the ability of synthetic agonists to control QS genes, we used a *qrr*4-*gfp* construct in an assay that has been described [31].

To assay *qrr*4 regulation of *cqsS*, the relative fluorescence of translational fusions (VCA0107B-GFP and CqsS*-*mKATE2) was measured in *E*. *coli* BWR1. *E*. *coli* carrying one of these plasmids also contained either an empty plasmid (pZA31) or pZA31 carrying anhydrous tetracycline-inducible *qrr*4. Qrr4-mediated repression of CqsS-mKATE2 was assessed. VCA0107-GFP is a known Qrr4-repressed target and was used as a positive control to show the system was functional. Overnight cultures were diluted 1:50 into fresh medium and aliquotted in triplicate into 96 well plates. Concentrations of anhydrous tetracycline from 0.4 to 100 ng/μl were added to induce *qrr*4 expression and the plates were incubated overnight at 30^°^C with shaking. OD_600_ and fluorescence were assessed using an Envision 2103 Multilabel Reader (Perkin Elmer).

### Preparation of CqsS inverted membrane vesicles

*E*. *coli* C43 (DE3) harboring pET21b plasmids carrying CqsS::6XHis or CqsS::6XHis D618N [17] were grown at 37^°^C with shaking in LB supplemented with kanamycin. Overnight cultures were diluted 1:100 in fresh LB with kanamycin and grown at 37^°^C with shaking for 3 h. Protein production was induced by the addition of 300 μM IPTG followed by growth overnight at 18^°^C with shaking. Cells were harvested by centrifugation at 5,000 *g* for 20 min, resuspended in lysis buffer (50 mM Tris pH 8.0, 200 mM NaCl, 5 mM MgCl_2_, complete mini EDTA-free protease inhibitor (Roche, #11836170001)), and lysed under 15,000 psi with a high-pressure pneumatic high shear fluid processor (Microfluidics, M-110Y). Cell lysates were clarified at 9,300 *g* for 30 min and the clarified supernatant was subjected to ultra-centrifugation at 180,000 *g* for 1 h. Membrane pellets were resuspended in kinase buffer (50 mM Tris pH 8.0, 100 mM KCl, 5 mM MgCl_2_, 10% (v/v) glycerol) and the CqsS proteins were quantified by western blot using CqsS::6XHis protein purified in detergent as the standard (see below). Phosphorylation assays were performed using inverted membrane vesicles containing 10 μM of WT CqsS or CqsS D618N protein as described [17].

### CqsS and LuxQ purification

To purify CqsS::3XFLAG protein, inverted membrane vesicles were prepared and following the ultra-centrifugation step, membrane pellets were resuspended in membrane extraction buffer (20 mM Tris pH 8, 100 mM NaCl, 20% (v/v) glycerol) and solubilized by rocking with 2% foscholine-12 (FC12: Avanti, #29557-51-5) at 4^°^C for 2 h. Solubilized membranes were incubated with M2 FLAG resin (Sigma-Aldrich, A2220) at 4^°^C for 2 h. Solubilized membranes were separated from the resin using gravity flow. The flow-through was passed over the resin twice more, and finally, the resin was washed with 10 column volumes of wash buffer (50 mM Tris pH 7.4, 150 mM NaCl, 0.2% FC12) and the protein was eluted with elution buffer (0.1 M glycine pH 3.5, 0.2% FC12). The eluted fractions were neutralized with 2% (v/v) 1 M Tris pH 8.0. Protein concentration was determined by Bradford assay (ThermoFisher, #23246), purity is shown in S12 Fig. LuxQ::3XFLAG was purified by the identical procedure.

CqsS::6XHis protein in detergent was purified as follows: Solubilized membranes were incubated with nickel resin (Qiagen, #30410) at 4^°^C for 2 h followed by separation from the resin using gravity flow. The flow-through was passed over the gravity column once again, and after that, the resin was washed with 10 column volumes of nickel binding buffer (20 mM Tris pH 8, 20 mM imidazole, 300 mM NaCl, 10% (v/v) glyercol, 4 mM 2-mercaptoethanol, 0.2% FC12) and the protein was eluted with 400 mM imidazole. Imidazole was removed by gel filtration on an S200 column with 20 mM Tris pH 8 buffer containing 150 mM NaCl, 5% (v/v) glycerol, 1 mM Tris-(2-carboxyethyl) phosphine (TCEP) and 0.2% FC12. Protein concentration was determined by Bradford assay (ThermoFisher, #23246).

### Quantitative immunoblotting

The number of CqsS dimers per *V*. *cholerae* cell was calculated by quantifying the concentration of CqsS per cell and converting it to dimer number. Cell density was determined by diluting and plating cultures on LB agar in triplicate. Aliquots of WT or mutant *V*. *cholerae* strains carrying CqsS::3XFLAG were subjected to centrifugation for 15 min at 8,000 *g* and the resulting pellets were flash frozen. The cells in the pellets were lysed for 10 min at 25^°^C by resuspending in 100 μl Bug Buster (Novagen, #70584–4) supplemented with 0.5% Triton-X, 50 μl/ml lysozyme, 25 U/mL benzonase, and 1 mM phenylmethylsulfonyl fluoride (PMSF) per 1.0 OD_600_ of pelleted culture. Protein from the cell lysate was solubilized with SDS-PAGE buffer for 1 h at 37^°^C. Various amounts of the samples were loaded onto 4–20% Mini-Protein TGX gels (Bio-Rad, #456–1096) alongside known amounts (0.06–2 ng) of detergent-purified CqsS::3XFLAG for comparison. The samples were electrophoresed for 1.5 h at 100 v. Proteins were transferred from the gels to PVDF membranes (Bio-Rad, #162–0174) for 1 h at 4^°^C at 100 v in 25 mM Tris buffer, 190 mM glycine, 20% methanol. Membranes were blocked for 1 h in 5% milk, washed 3 times with TBST (140 mM NaCl, 20 mM Tris-HCl pH 7.6, 0.1% Tween) and incubated for 1 h with 0.2 μg/ml monoclonal Anti-FLAG-Peroxidase antibody (Sigma, A8592) in TBST with 3% BSA (Sigma, A3059). After washing three times with TBST, membranes were exposed using the Amersham ECL western blotting detection reagent (GE Healthcare, RPN2106) for 40 s. Linear regression analysis of band intensity (volume) of detergent-purified CqsS::3XFLAG was performed for each gel to calculate the amount of CqsS in the band from the *V*. *cholerae* samples. Protein amount was converted to molarity using the MW of the CqsS::3XFLAG construct of 83.61 kDa. The CqsS concentration and the cell density of each *V*. *cholerae* sample were used to estimate CqsS dimers per cell.

### qRT-PCR analyses

*V*. *cholerae* overnight cultures were back-diluted 1:100 into fresh medium and grown to OD_600_ = 2.0. Autoinducers were added at the indicated concentrations at the time of dilution. RNA was harvested from three independent cultures using the RNeasy mini kit (Qiagen, #4104) and cDNA was generated as described [73] with SuperScript III reverse transcriptase (Invitrogen, #18080–044) using 1.5 μg of RNA. Real-time PCR analyses were performed as described [73] using a QuantStudio 6 Flex Real-Time PCR detection system (ThermoFisher) and the Sybr Green mix (Quanta, #95074). Quadruplicate technical replicates for each experiment were analyzed by a comparative C_T_ method (Applied Biosystems) in which the relative amount of *hapA* [74] was normalized to the internal *hfq* control RNA to determine the relative RNA levels. *hapA* transcript levels in *V*. *cholerae* mutants were normalized to WT levels.

## Supporting information

S1 Data Numerical data for Figs 2A, 2C, 2D, 3, 4, 5, 6B, 6E and 7. (XLSX)

S2 Data Numerical data for S1, S3C, S4, S5, S6, S7, S8, S9 and S10 Figs. (XLSX)

S1 Table Strains used in this study. (TIFF)

S2 Table Primers used in this study. (TIFF)

S1 Fig CqsS::3XFLAG and LuxQ::3XFLAG are functional. Light production from a *V*. *cholerae* strain lacking all QS receptors (WN3360: Δ*luxQ* Δ*cqsS* Δ*vpsS* Δ*cqsR*, black squares in both panels) compared to *V*. *cholerae* strains with the gene encoding the following receptor at its endogenous location in the chromosome: A) CqsS (WN3651, black circles) or CqsS::3XFLAG (AH408, white circles) or B) LuxQ (WN3649, black circles) or LuxQ::3XFLAG (AH403, white circles). All strains carry the QS-controlled *luxCDABE* operon. Relative light units (RLU) are defined as counts/min ml^-1^ per OD_600_. The figure shows representative data from n = 3 experiments.
(TIFF)

S2 Fig CqsS::3XFLAG and LuxQ::3XFLAG protein levels over growth. Representative western blot showing the amounts of CqsS::3XFLAG and LuxQ::3XFLAG in *V*. *cholerae* cells (strain AH420) collected at the specified OD_600_ in three independent experiments. Lysate from 0.06 OD_600_ of cells was loaded per well.
(TIFF)

S3 Fig Levels of CqsS::3XFLAG protein in WT *V*. *cholerae* and QS mutants at LCD. A and B) Representative quantitative western blots showing the indicated amounts of purified CqsS protein (left-most lanes) and the CqsS present in the specified *V*. *cholerae* strains at OD_600_ = 0.2. To assess CqsS levels in cells, lysate from 0.06 OD_600_ of cells was loaded per well. C) Relative CqsS dimers per cell in the *V*. *cholerae* strains from panel B at LCD (OD_600_ = 0.2) normalized to WT levels. Experiments were performed in quadruplicate and error bars represent standard errors of the mean.
(TIFF)

S4 Fig Qrr4 indirectly regulates *cqsS*. Fluorescence from plasmid-encoded VCA0107-GFP (circles) [24] and CqsS-mKATE2 (squares) was measured in *E*. *coli*. Either an empty vector (pZA31-*luc*NB, black) or pZA31-*luc*NB carrying tetracycline-inducible *qrr*4 (pYS245, red) [24] was present in each strain. *VCA0107* encodes a type VI secretion system component that is regulated by Qrr4. Data represent two experiments conducted in triplicate. Error bars represent standard errors of the mean.
(TIFF)

S5 Fig Synthetic CAI-1 standard curve used to quantify CAI-1 concentrations in *V*. *cholerae* cell-free culture fluids. Representative bioluminescence output from the *V*. *cholerae* CAI-1 reporter strain WN1102 in response to the indicated amounts of synthetic CAI-1. The CAI-1 concentration in cell-free culture fluids was extrapolated using the Prism software calculation log (agonist) vs. response. Relative light units (RLU) are defined as counts/min ml^-1^ per OD_600_.
(TIFF)

S6 Fig CqsS receptor occupancy at different CAI-1 concentrations. A) The receptor occupancy equation used to calculate the amount of CqsS bound by CAI-1 with R_L_ (ligand-bound receptor), R_TOT_ (total receptor), L (ligand concentration) and K_d_. B) Theoretical ratio of bound CqsS receptor as a function of CAI-1 concentration. The K_d_ for CAI-1 binding to CqsS has been reported to be 35 nM [31]. We verified this finding (see Fig 6 of the main text): we measured the K_d_ to be 38 nM and we used that value for the calculation shown in this plot and for all calculations in the main text.
(TIFF)

S7 Fig Effect of autoinducers on CqsS protein levels. A) Light production from the *V*. *cholerae* double synthase mutant (AH371: Δ*cqsA* Δ*luxS*, carries CqsS::3XFLAG) harboring the *luxCDABE* operon is shown in response to increasing concentrations of CAI-1 (red) and AI-2 (black). This experiment was performed in triplicate and standard errors of the mean, albeit small, are shown. Relative light units (RLU) are defined as counts/min ml^-1^ per OD_600_. B) Representative western blot showing CqsS levels in WT (AH330, carries CqsS::3XFLAG) *V*. *cholerae* and the Δ*cqsA* Δ*luxS* strain AH371 (carries CqsS::3XFLAG) at OD_600_ = 0.2 (LCD) and 2.0 (HCD). AI-2 and CAI-1 were provided at 1 μM and 5 μM, respectively. Twice as much LCD than HCD lysate was loaded to ensure band intensities were within the linear range of detection.
(TIFF)

S8 Fig CAI-1 is more effective than AI-2 in repression of Qrr4. Bioluminescence from a *qrr*4-*luxCDABE* transcriptional fusion in WT (black) and in Δ*cqsA* Δ*luxS* (white) *V*. *cholerae*, the latter in the presence of AI-2 (blue), CAI-1 (red) or both autoinducers (purple) at saturating 1 μM and 5 μM concentrations, respectively. The experiment was performed in triplicate and standard errors of the mean are shown.
(TIFF)

S9 Fig The *V*. *cholerae* QS response to AI-2 increases when LuxPQ is the dominant receptor. A) Relative receptor ratios in *V*. *cholerae* cells carrying CqsS::3XFLAG and LuxQ::3XFLAG when *cqsS* is driven by its endogenous promoter, P_*cqsS*_-CqsS::3XFLAG (strain AH420, black), and when *cqsS* is driven by the *luxPQ* promoter, P_*luxPQ*_-CqsS::3XFLAG (AH466, white). Data show duplicate samples and error bars represent standard errors of the mean. B) Bioluminescence output from the QS-controlled *luxCDABE* operon in WT *V*. *cholerae* (AH330: carries *P*_*cqsS*_*-*CqsS::3XFLAG, black) and in the double Δ*cqsA* Δ*luxS* autoinducer synthase mutant with *cqsS* driven by the *luxPQ* promoter (AH468: Δ*cqsA* Δ*luxS*, carries P_*luxPQ*_-CqsS::3XFLAG, white) in response to AI-2 (blue), CAI-1 (red), or both AI-2 and CAI-1 (purple) at saturating 1 μM and 5 μM concentrations, respectively. Relative light units (RLU) are defined as counts/min ml^-1^ per OD_600_.
(TIFF)

S10 Fig Compound #1 requires the tetrazole side chain for CqsS agonist activity. Light-production from the *V*. *cholerae* CAI-1 reporter strain WN1102 is shown in response to increasing concentrations of CAI-1 (red), compound #1 (black) and compound #6 (white). See Fig 6 of the main text for structures. RLU are defined as counts/min ml^-1^ per OD_600_. This experiment was performed in triplicate and standard errors of the mean, albeit small, are shown.
(TIFF)

S11 Fig Ligand-directed inhibition of CqsS and CqsS D618N autophosphorylation. A) Representative CqsS D618N autophosphorylation assays in the presence of DMSO or the indicated amounts of CAI-1, compound #1, or compound #10. In each gel, CqsS D618N~P band intensities in the lanes with additions of 1 μM of CAI-1, #1, and #10 were quantified and normalized to the average band intensities when DMSO was added. Data are presented in Fig 7A of the main text. B) Representative autophosphorylation of WT CqsS in the presence of DMSO or the indicated amounts of CAI-1, compound #1, and compound #10. In each gel, band intensities for CqsS~P following addition of CAI-1, #1, or #10 were quantified and normalized to the CqsS~P in the presence of DMSO. The titration data are presented in Fig 7B of the main text. In both panels, the minus symbols above each gel denote duplicates of the DMSO controls.
(TIFF)

S12 Fig Detergent-purified CqsS protein. Coomaisse stained gel to assess protein purity. Lane 1, PageRuler Plus protein ladder, Lane 2, 2 µg of purified CqsS::3XFLAG protein.
(TIFF)
