# Supplementary figures and images for: Asymmetric regulation of quorum-sensing receptors drives autoinducer-specific gene expression programs in Vibrio cholerae
Source: PLoS Genet. 2017 May 26;13(5):e1006826. doi: 10.1371/journal.pgen.1006826 (PMC5467912; doi:10.1371/journal.pgen.1006826)

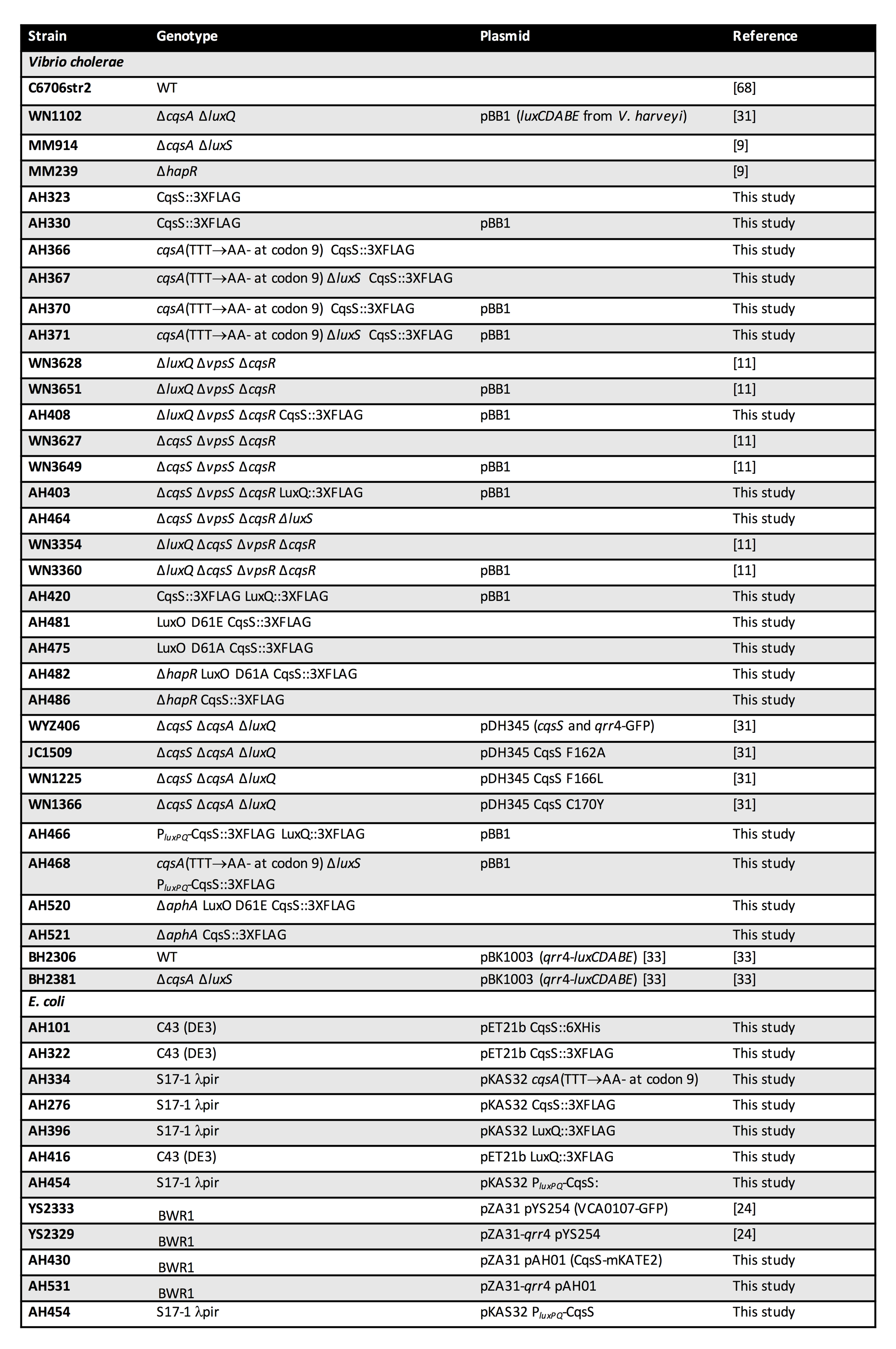

Supplement: S1 Table — (TIFF) [file pgen.1006826.s003.tiff]

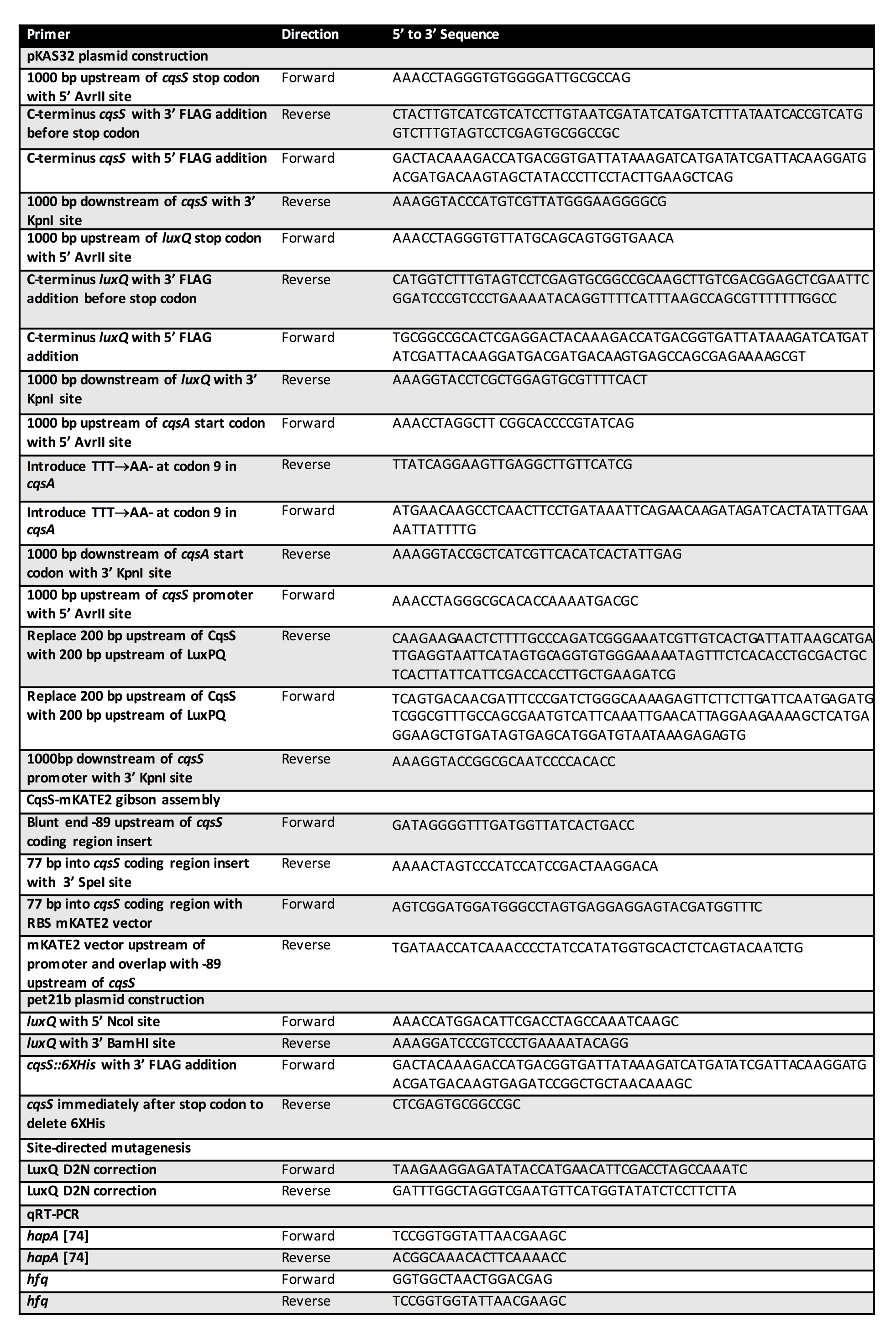

Supplement: S2 Table — (TIFF) [file pgen.1006826.s004.tiff]

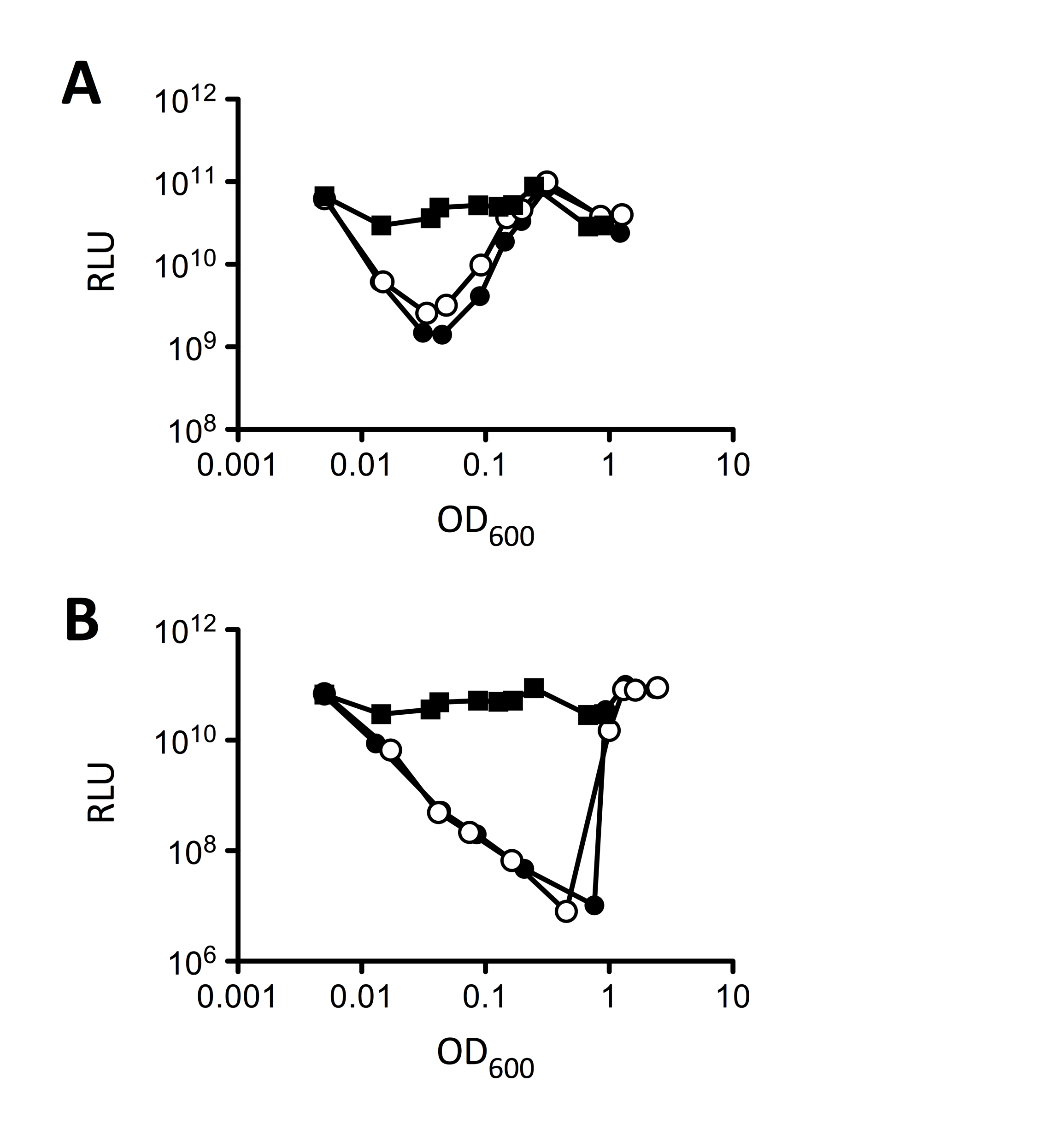

Supplement: S1 Fig — Light production from a V. cholerae strain lacking all QS receptors (WN3360: ΔluxQ ΔcqsS ΔvpsS ΔcqsR, black squares in both panels) compared to V. cholerae strains with the gene encoding the following receptor at its endogenous location in the chromosome: A) CqsS (WN3651, black circles) or CqsS::3XFLAG (AH408, white circles) or B) LuxQ (WN3649, black circles) or LuxQ::3XFLAG (AH403, white circles). All strains carry the QS-controlled luxCDABE operon. Relative light units (RLU) are defined as counts/min ml-1 per OD600. The figure shows representative data from n = 3 experiments. (TIFF) [file pgen.1006826.s005.tiff]

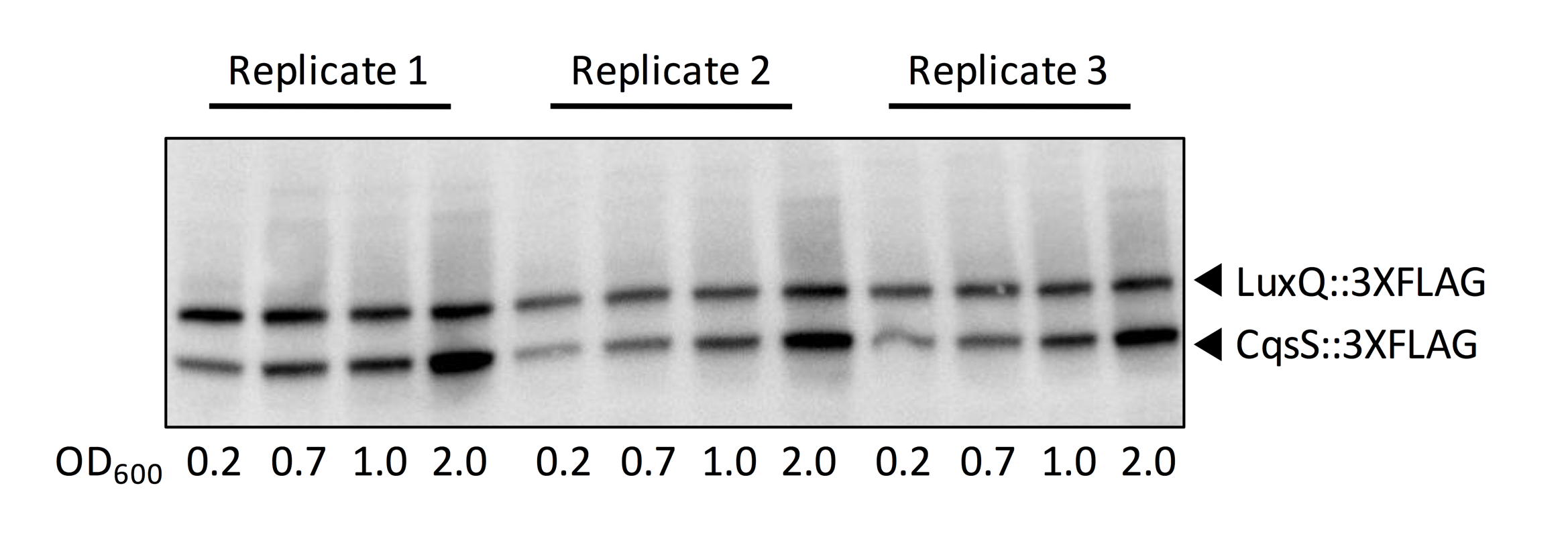

Supplement: S2 Fig — Representative western blot showing the amounts of CqsS::3XFLAG and LuxQ::3XFLAG in V. cholerae cells (strain AH420) collected at the specified OD600 in three independent experiments. Lysate from 0.06 OD600 of cells was loaded per well. (TIFF) [file pgen.1006826.s006.tiff]

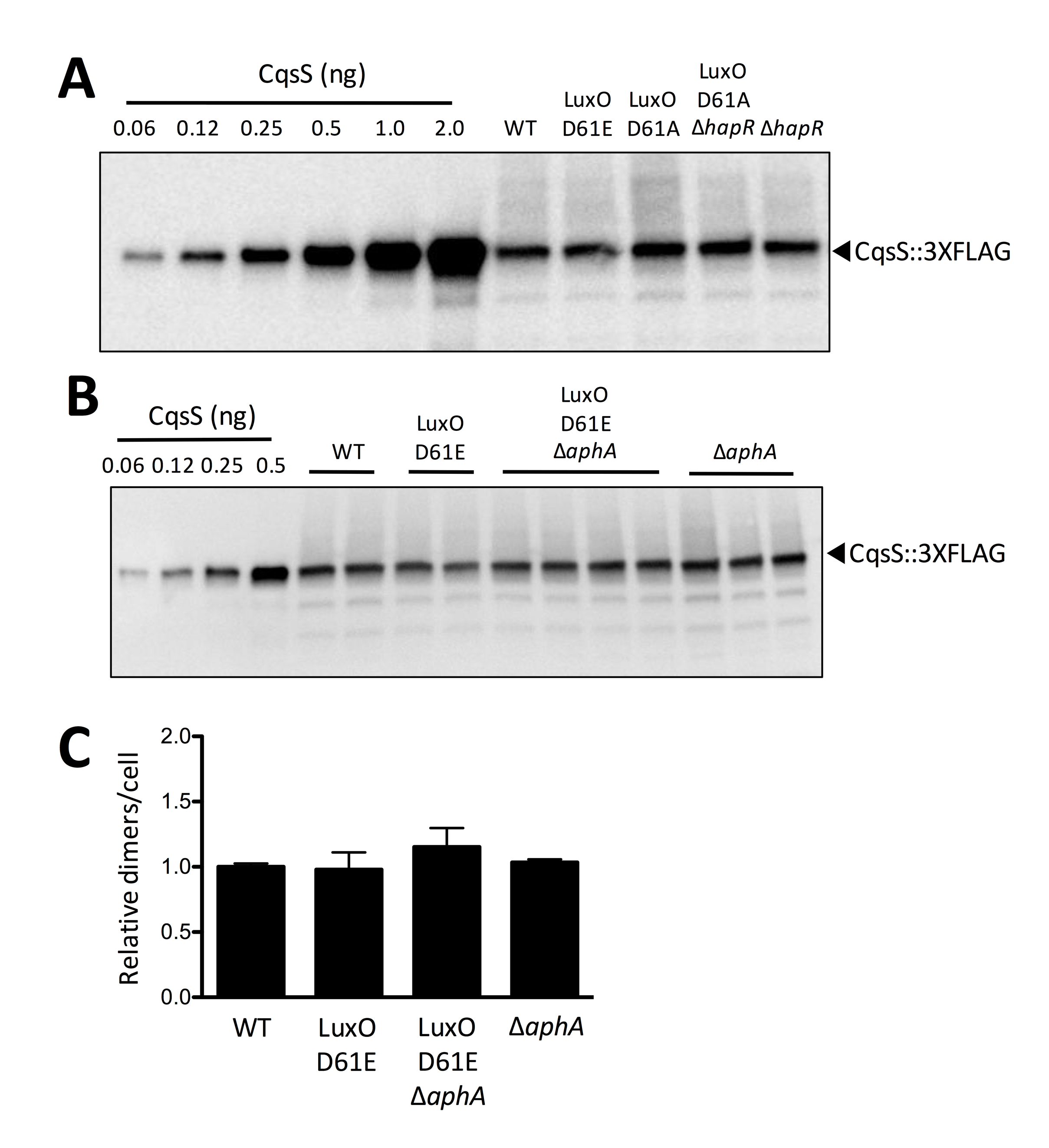

Supplement: S3 Fig — A and B) Representative quantitative western blots showing the indicated amounts of purified CqsS protein (left-most lanes) and the CqsS present in the specified V. cholerae strains at OD600 = 0.2. To assess CqsS levels in cells, lysate from 0.06 OD600 of cells was loaded per well. C) Relative CqsS dimers per cell in the V. cholerae strains from panel B at LCD (OD600 = 0.2) normalized to WT levels. Experiments were performed in quadruplicate and error bars represent standard errors of the mean. (TIFF) [file pgen.1006826.s007.tiff]

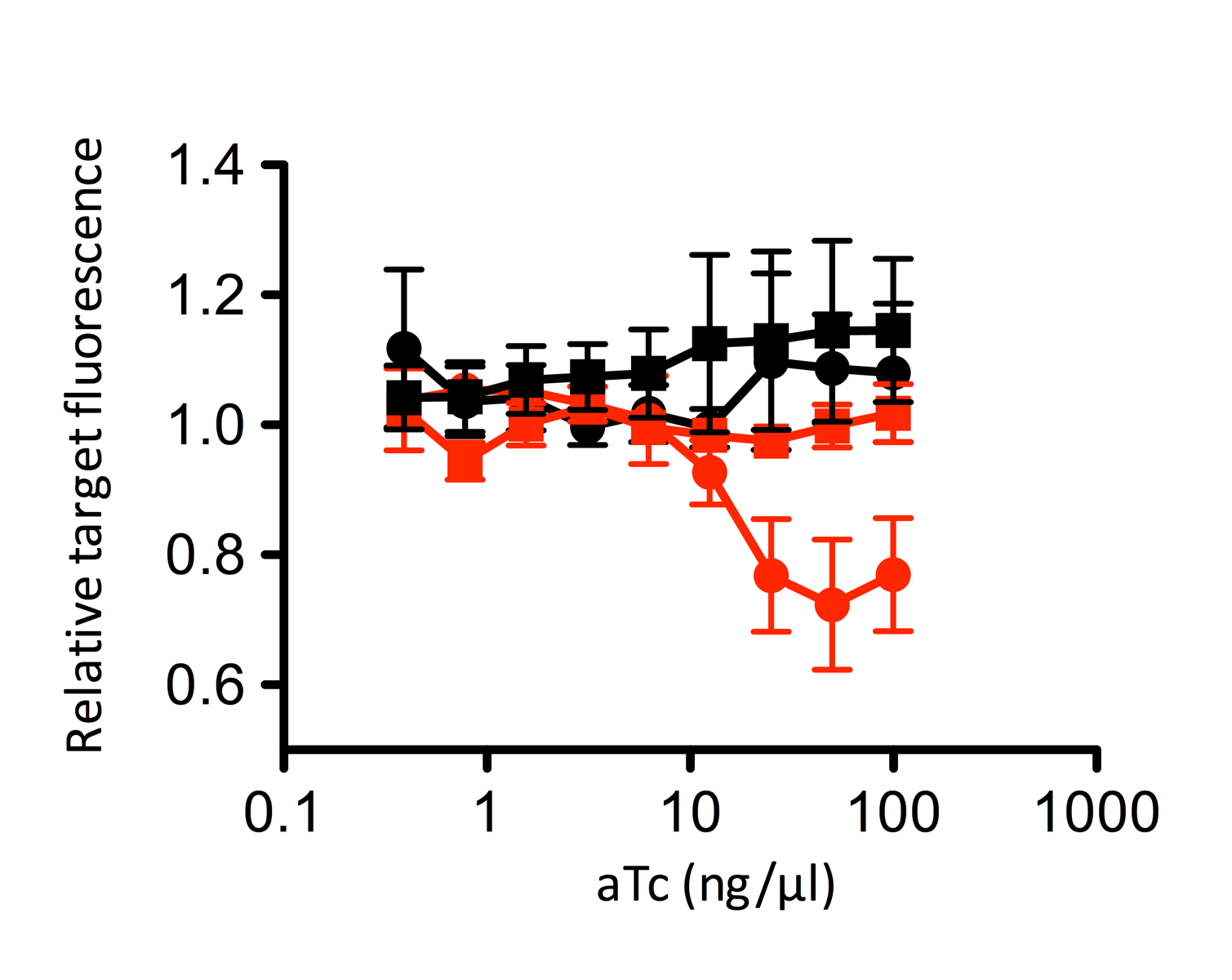

Supplement: S4 Fig — Fluorescence from plasmid-encoded VCA0107-GFP (circles) [24] and CqsS-mKATE2 (squares) was measured in E. coli. Either an empty vector (pZA31-lucNB, black) or pZA31-lucNB carrying tetracycline-inducible qrr4 (pYS245, red) [24] was present in each strain. VCA0107 encodes a type VI secretion system component that is regulated by Qrr4. Data represent two experiments conducted in triplicate. Error bars represent standard errors of the mean. (TIFF) [file pgen.1006826.s008.tiff]

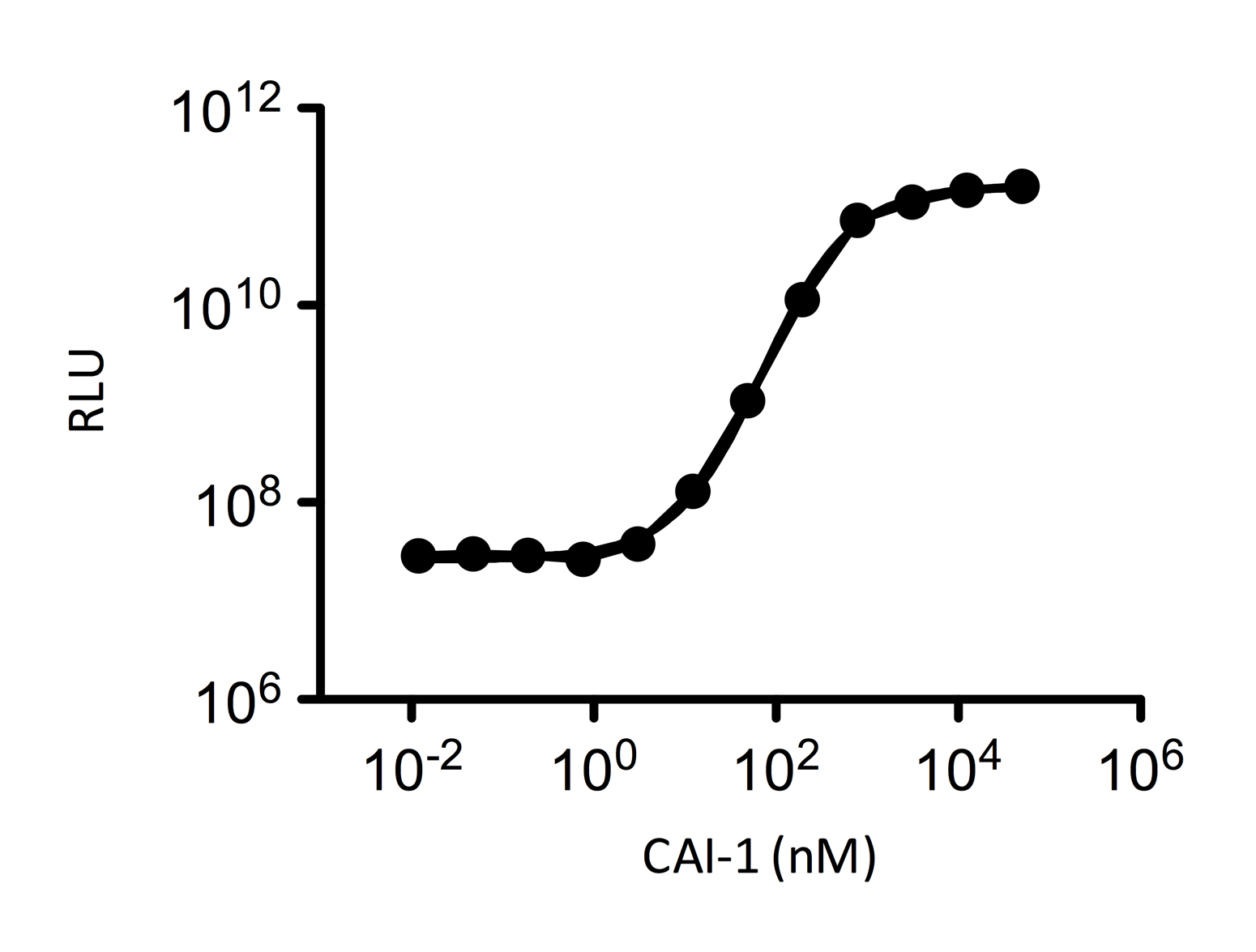

Supplement: S5 Fig — Representative bioluminescence output from the V. cholerae CAI-1 reporter strain WN1102 in response to the indicated amounts of synthetic CAI-1. The CAI-1 concentration in cell-free culture fluids was extrapolated using the Prism software calculation log (agonist) vs. response. Relative light units (RLU) are defined as counts/min ml-1 per OD600. (TIFF) [file pgen.1006826.s009.tiff]

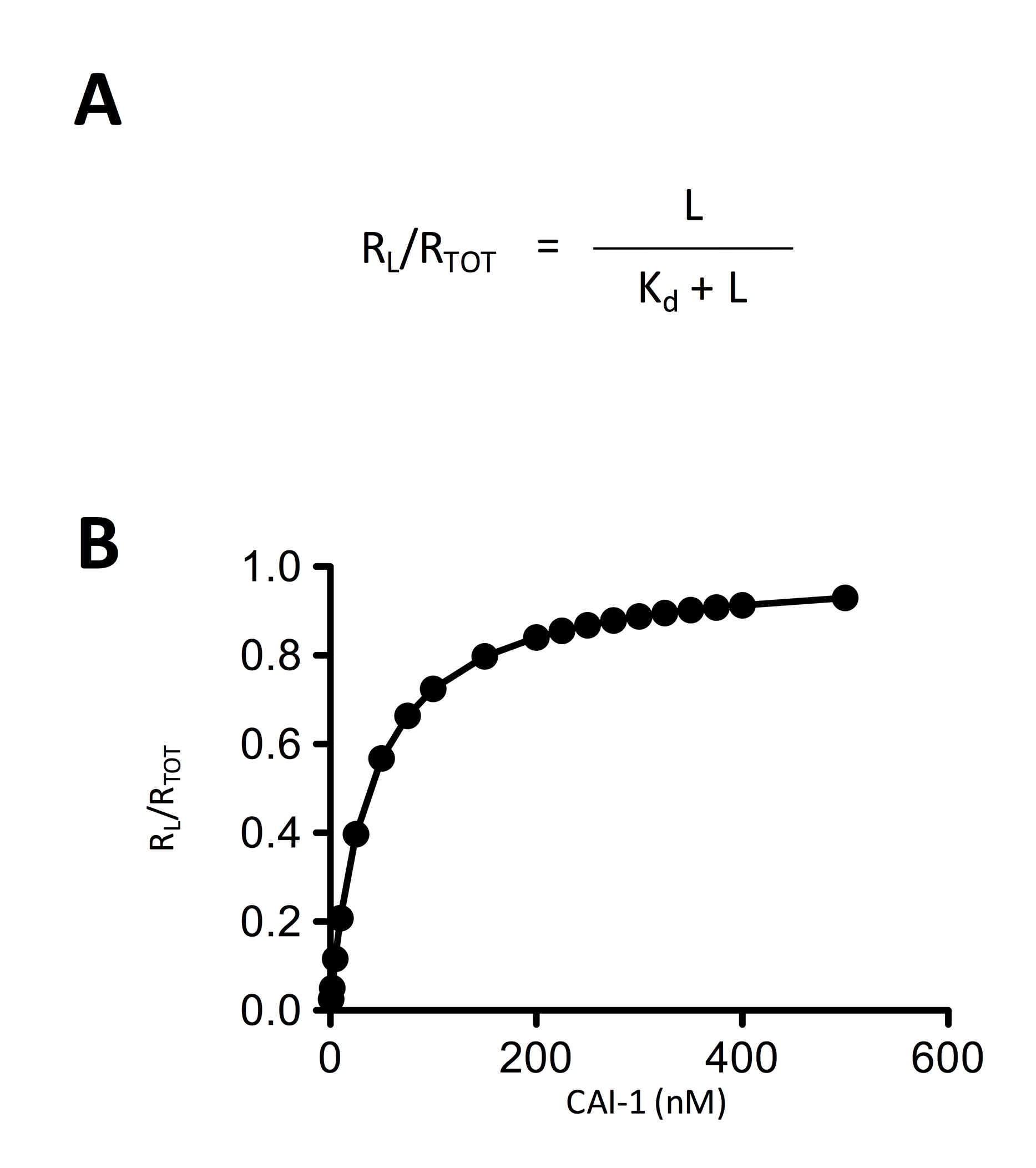

Supplement: S6 Fig — A) The receptor occupancy equation used to calculate the amount of CqsS bound by CAI-1 with RL (ligand-bound receptor), RTOT (total receptor), L (ligand concentration) and Kd. B) Theoretical ratio of bound CqsS receptor as a function of CAI-1 concentration. The Kd for CAI-1 binding to CqsS has been reported to be 35 nM [31]. We verified this finding (see Fig 6 of the main text): we measured the Kd to be 38 nM and we used that value for the calculation shown in this plot and for all calculations in the main text. (TIFF) [file pgen.1006826.s010.tiff]

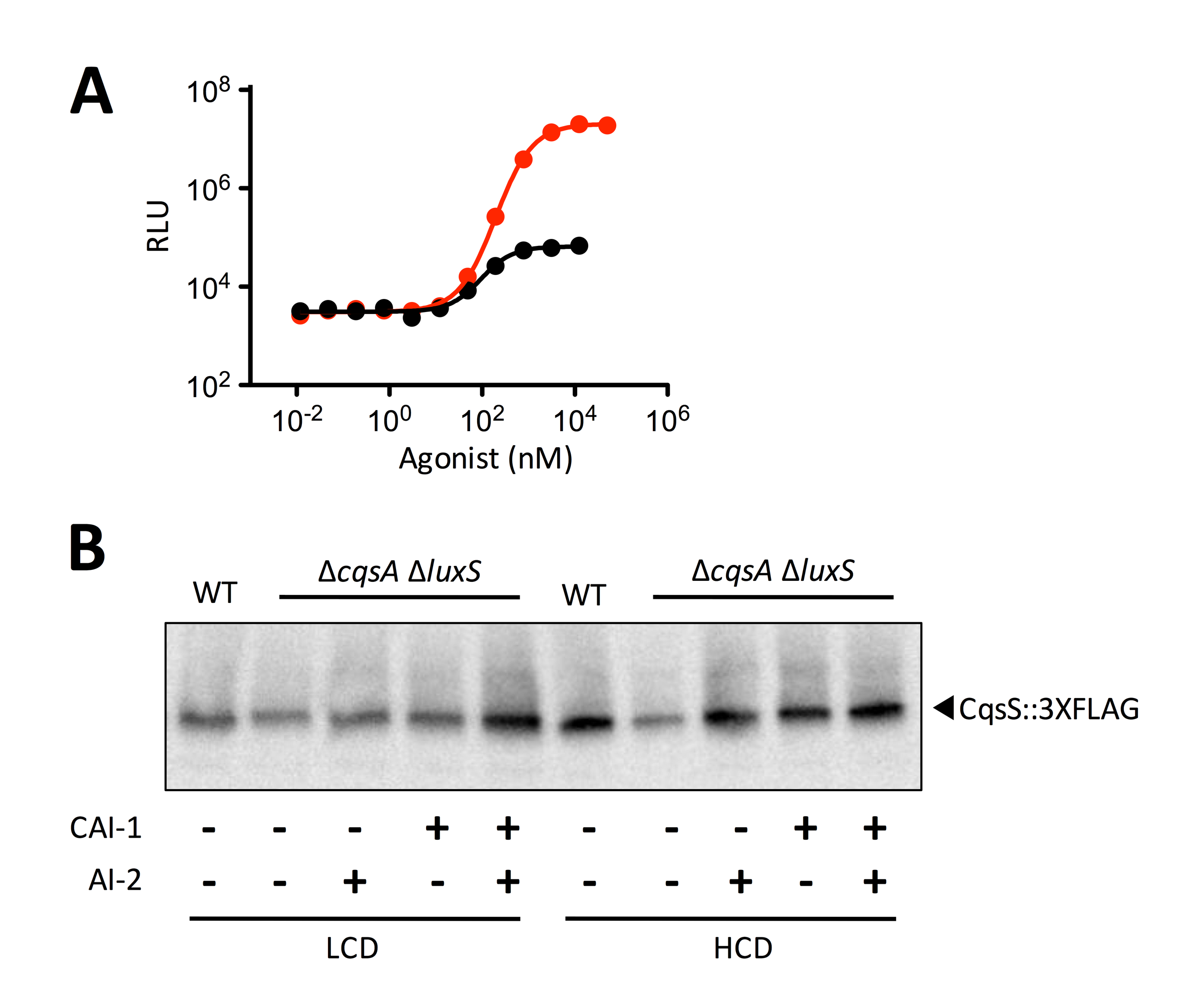

Supplement: S7 Fig — A) Light production from the V. cholerae double synthase mutant (AH371: ΔcqsA ΔluxS, carries CqsS::3XFLAG) harboring the luxCDABE operon is shown in response to increasing concentrations of CAI-1 (red) and AI-2 (black). This experiment was performed in triplicate and standard errors of the mean, albeit small, are shown. Relative light units (RLU) are defined as counts/min ml-1 per OD600. B) Representative western blot showing CqsS levels in WT (AH330, carries CqsS::3XFLAG) V. cholerae and the ΔcqsA ΔluxS strain AH371 (carries CqsS::3XFLAG) at OD600 = 0.2 (LCD) and 2.0 (HCD). AI-2 and CAI-1 were provided at 1 μM and 5 μM, respectively. Twice as much LCD than HCD lysate was loaded to ensure band intensities were within the linear range of detection. (TIFF) [file pgen.1006826.s011.tiff]

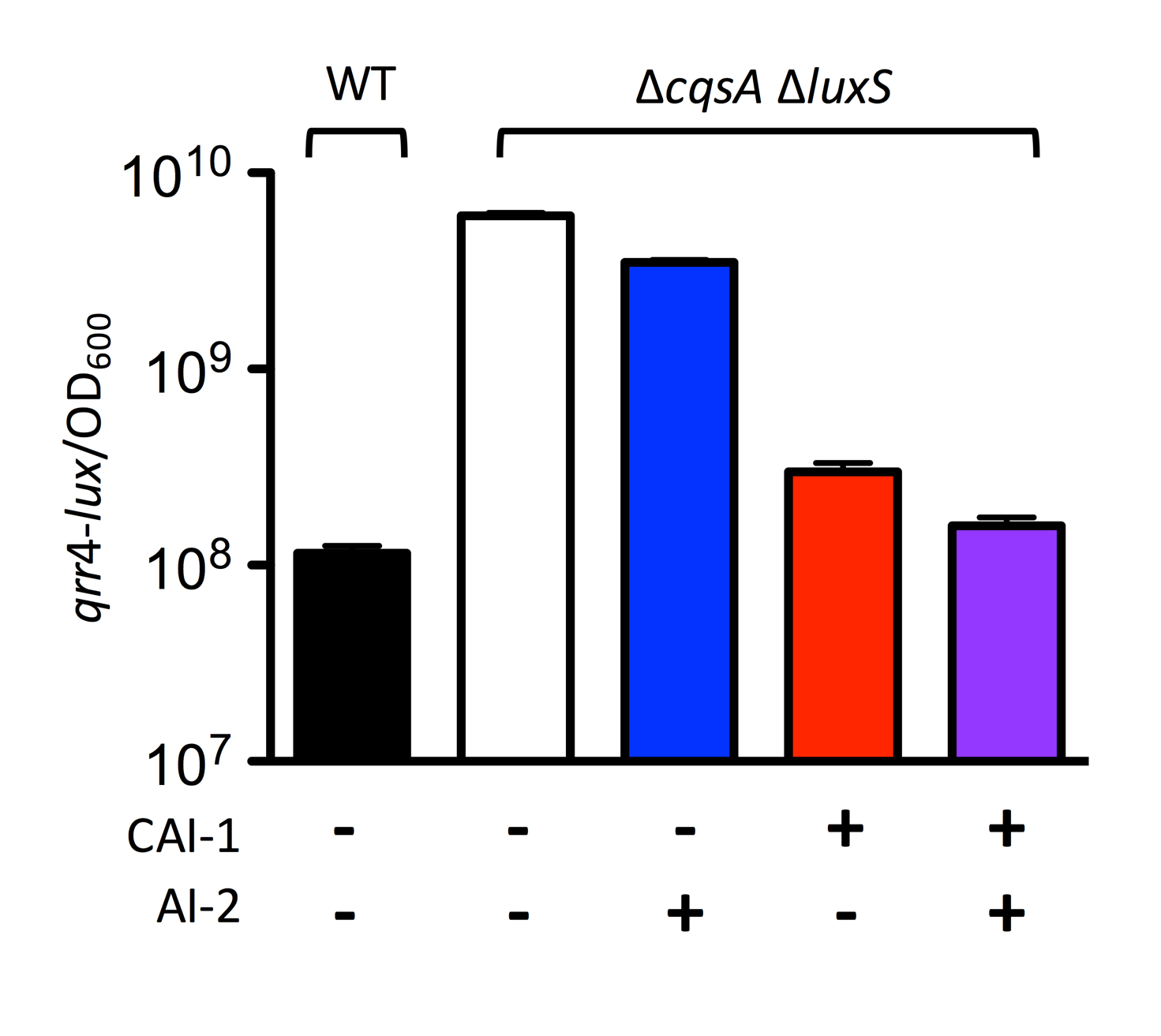

Supplement: S8 Fig — Bioluminescence from a qrr4-luxCDABE transcriptional fusion in WT (black) and in ΔcqsA ΔluxS (white) V. cholerae, the latter in the presence of AI-2 (blue), CAI-1 (red) or both autoinducers (purple) at saturating 1 μM and 5 μM concentrations, respectively. The experiment was performed in triplicate and standard errors of the mean are shown. (TIFF) [file pgen.1006826.s012.tiff]

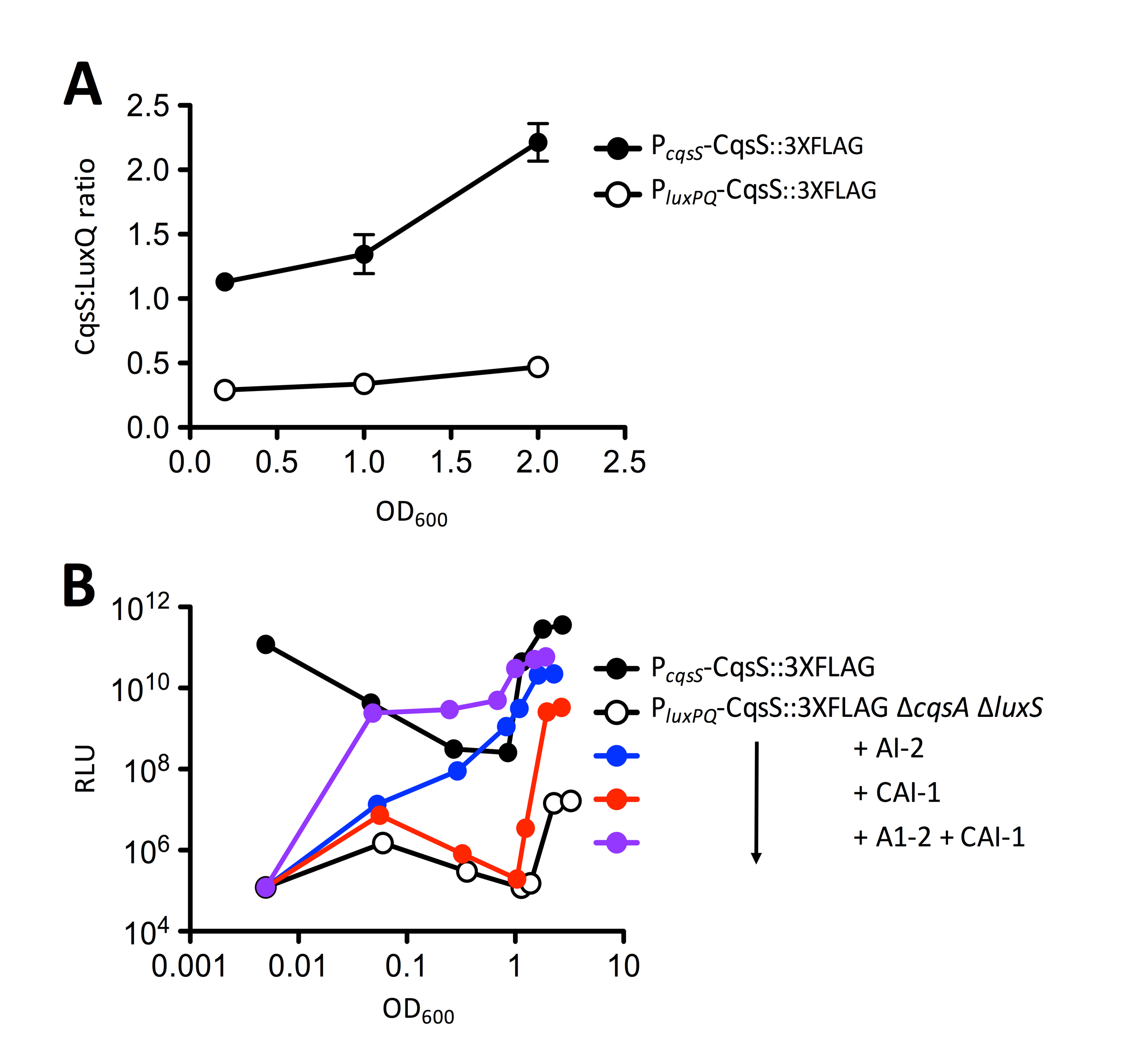

Supplement: S9 Fig — A) Relative receptor ratios in V. cholerae cells carrying CqsS::3XFLAG and LuxQ::3XFLAG when cqsS is driven by its endogenous promoter, PcqsS-CqsS::3XFLAG (strain AH420, black), and when cqsS is driven by the luxPQ promoter, PluxPQ-CqsS::3XFLAG (AH466, white). Data show duplicate samples and error bars represent standard errors of the mean. B) Bioluminescence output from the QS-controlled luxCDABE operon in WT V. cholerae (AH330: carries PcqsS-CqsS::3XFLAG, black) and in the double ΔcqsA ΔluxS autoinducer synthase mutant with cqsS driven by the luxPQ promoter (AH468: ΔcqsA ΔluxS, carries PluxPQ-CqsS::3XFLAG, white) in response to AI-2 (blue), CAI-1 (red), or both AI-2 and CAI-1 (purple) at saturating 1 μM and 5 μM concentrations, respectively. Relative light units (RLU) are defined as counts/min ml-1 per OD600. (TIFF) [file pgen.1006826.s013.tiff]

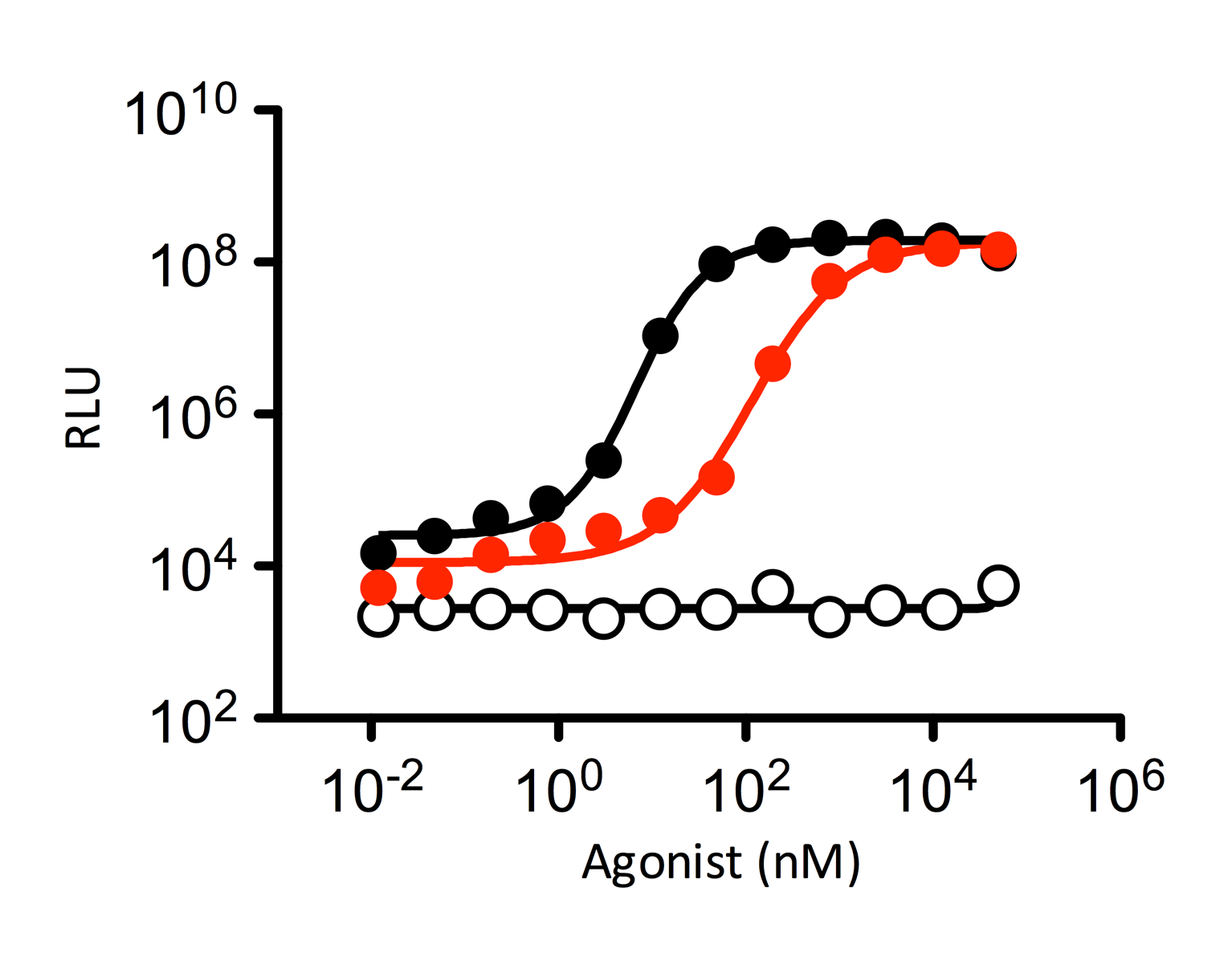

Supplement: S10 Fig — Light-production from the V. cholerae CAI-1 reporter strain WN1102 is shown in response to increasing concentrations of CAI-1 (red), compound #1 (black) and compound #6 (white). See Fig 6 of the main text for structures. RLU are defined as counts/min ml-1 per OD600. This experiment was performed in triplicate and standard errors of the mean, albeit small, are shown. (TIFF) [file pgen.1006826.s014.tiff]

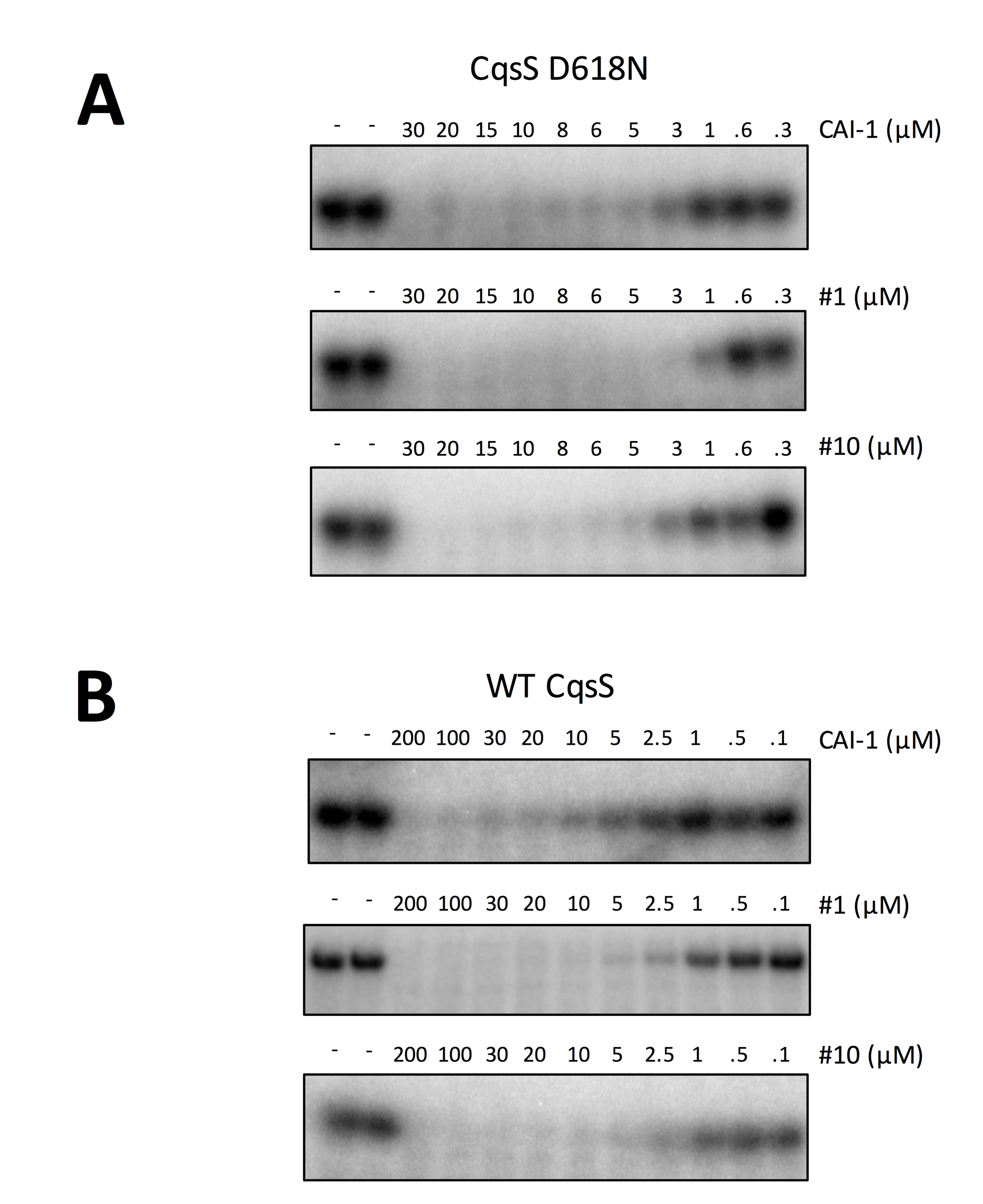

Supplement: S11 Fig — A) Representative CqsS D618N autophosphorylation assays in the presence of DMSO or the indicated amounts of CAI-1, compound #1, or compound #10. In each gel, CqsS D618N~P band intensities in the lanes with additions of 1 μM of CAI-1, #1, and #10 were quantified and normalized to the average band intensities when DMSO was added. Data are presented in Fig 7A of the main text. B) Representative autophosphorylation of WT CqsS in the presence of DMSO or the indicated amounts of CAI-1, compound #1, and compound #10. In each gel, band intensities for CqsS~P following addition of CAI-1, #1, or #10 were quantified and normalized to the CqsS~P in the presence of DMSO. The titration data are presented in Fig 7B of the main text. In both panels, the minus symbols above each gel denote duplicates of the DMSO controls. (TIFF) [file pgen.1006826.s015.tiff]

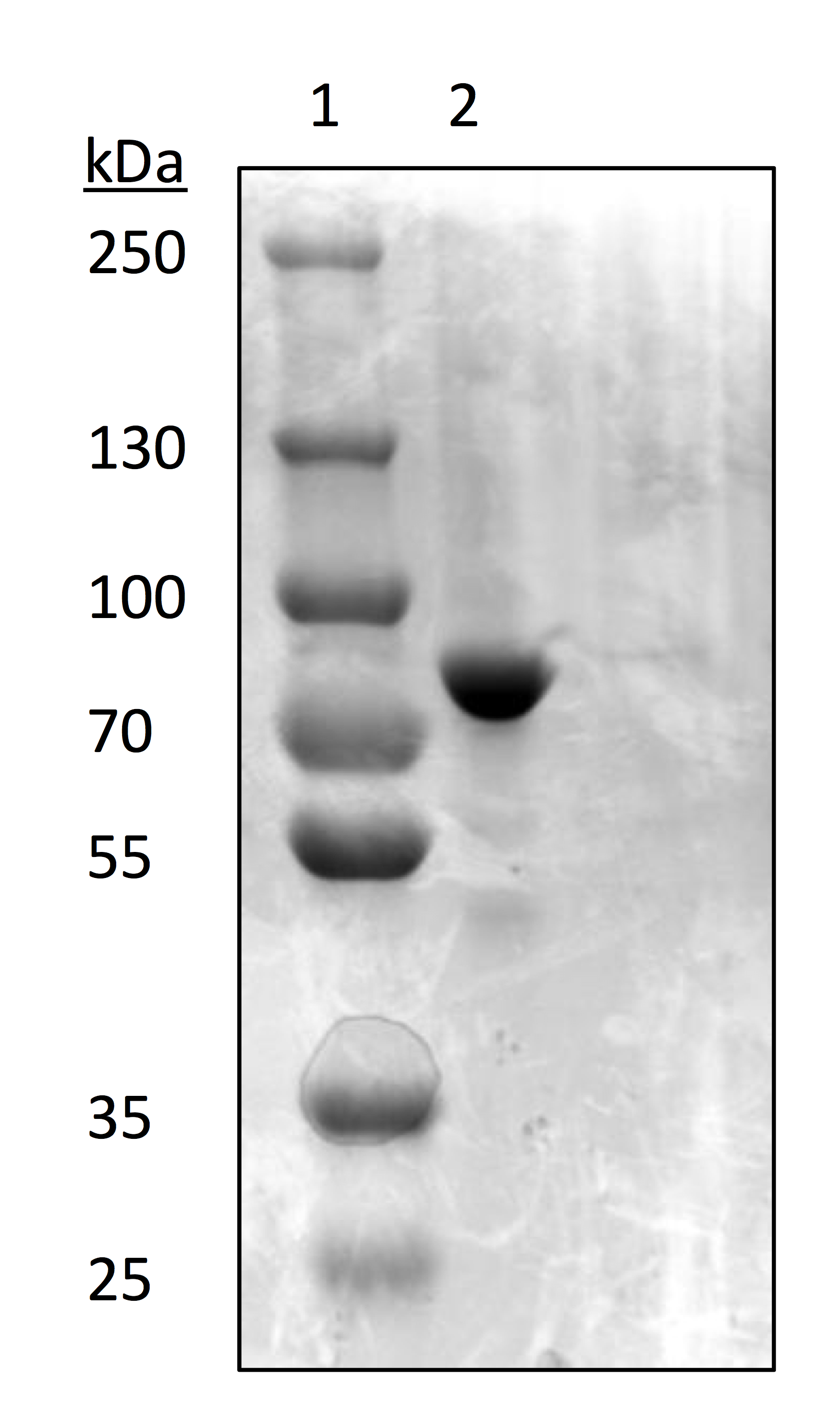

Supplement: S12 Fig — Coomaisse stained gel to assess protein purity. Lane 1, PageRuler Plus protein ladder, Lane 2, 2 µg of purified CqsS::3XFLAG protein. (TIFF) [file pgen.1006826.s016.tiff]
